# A Cost‐Effective Hemin‐Based Artificial Enzyme Allows for Practical Applications

**DOI:** 10.1002/advs.202402237

**Published:** 2024-06-25

**Authors:** Dehui Qiu, Fangni He, Yuan Liu, Zhaoxi Zhou, Yuqin Yang, Zhongwen Long, Qianqian Chen, Desheng Chen, Shijiong Wei, Xuanxiang Mao, Xiaobo Zhang, Jean‐Louis Mergny, David Monchaud, Huangxian Ju, Jun Zhou

**Affiliations:** ^1^ State Key Laboratory of Analytical Chemistry for Life Science School of Chemistry and Chemical Engineering Nanjing University Nanjing 210023 China; ^2^ Laboratoire d'Optique et Biosciences (LOB) Ecole Polytechnique CNRS INSERM Institut Polytechnique de Paris Palaiseau 91120 France; ^3^ Institut de Chimie Moléculaire (ICMUB), CNRS UMR6302, UBFC Dijon 21078 France

**Keywords:** artificial enzyme, G‐quadruplex, hemin, histidine analogs

## Abstract

Nanomaterials excel in mimicking the structure and function of natural enzymes while being far more interesting in terms of structural stability, functional versatility, recyclability, and large‐scale preparation. Herein, the story assembles hemin, histidine analogs, and G‐quadruplex DNA in a catalytically competent supramolecular assembly referred to as assembly‐activated hemin enzyme (AA‐heminzyme). The catalytic properties of AA‐heminzyme are investigated both in silico (by molecular docking and quantum chemical calculations) and in vitro (notably through a systematic comparison with its natural counterpart horseradish peroxidase, HRP). It is found that this artificial system is not only as efficient as HRP to oxidize various substrates (with a turnover number *k*_cat_ of 115 s^−1^) but also more practically convenient (displaying better thermal stability, recoverability, and editability) and more economically viable, with a catalytic cost amounting to <10% of that of HRP. The strategic interest of AA‐heminzyme is further demonstrated for both industrial wastewater remediation and biomarker detection (notably glutathione, for which the cost is decreased by 98% as compared to commercial kits).

## Graphical abstract

A nanomaterial comprising hemin, histidine analogs, and G‐quadruplex (G4)‐forming sequences is assembled and its peroxidase‐like enzymatic activity is investigated. The resulting catalytic nanoparticles, referred to as assembly‐activated hemin enzyme (AA‐heminzyme), are readily synthesized, at low cost, exhibit excellent catalytic activity, heat resistance, and recyclability, and are found quite active in two representative applications.

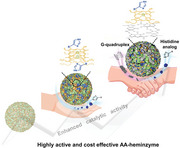

## 1 Introduction

Artificial enzymes, especially peroxidase mimics, have been widely used in biosensing, cancer therapy, environmental protection and food safety.^[^ ^1^ ^]^ For replacing natural enzymes, artificial enzymes should meet key requirements: they must reach the level of catalytic activities of corresponding native enzymes while being more practically convenient (usable at high temperatures, high/low pH, etc.), and must also be less expensive than their native counterparts. Hemin, an inexpensive iron porphyrin derivative, has been extensively used to construct peroxidase‐mimicking artificial catalytic systems,^[^ ^2^ ^]^ a quite active field of research because of the numerous applications of peroxidases for biosensing purposes. Hemin is particularly used in combination with G‐quadruplex DNA (or G4 DNA), a thermodynamically stable higher‐order DNA structure:^[^ ^3^ ^]^ the resulting G4/hemin systems, known as G4/hemin DNAzymes or G4‐DNAzymes,^[^ ^4^ ^]^ are currently being exploited in biocatalysis owing to their exquisite properties in terms of versatility, designability and addressability.^[^ ^5^ ^]^ When included in nanomaterials, hemin‐based systems rely on the combination of hemin with carbon nanotubes, graphene, hydrogels, polymers, proteins,^[^ ^6^ ^]^ and peptides^[^ ^7^ ^]^ to address some of the hemin classical issues, that is, its self‐aggregation deactivation and low solubility in aqueous solutions. However, the catalytic activities of these nanomaterials still lag far behind natural enzymes notably the reference enzyme horseradish peroxidase (HRP), which represents a severe limitation to their wider use, a limitation that must be urgently overcome.

The unique properties of HRP originate from its interaction with a heme cofactor, driven by the presence of a well‐defined network of amino acid residues in its catalytic center,^[^ ^8^ ^]^ which highlights the importance of the heme microenvironment. However, HRP suffers from several known limitations, notably related to its biosourced origin that requires an expensive production that furthermore leads to mixtures of HRP isoforms responsible for batch‐to‐batch variations^[^ ^9^ ^]^ and poorly reproducible results.^[^ ^10^ ^]^ Nanomaterial‐based mimetic enzymes are a reliable alternative to the use of HRP, as their synthesis and characterizations are controlled, which ensures a better reproducibility. In this context, we recently reported on a very active system named chimeric peptide DNAzyme, or CPDzyme, in which we recapitulated such a peptide/DNA microenvironment to provide an artificial enzyme displaying a higher turnover number (*k*_cat_) than HRP,^[^ ^2b^ ^]^ in addition to be more chemically robust. The only sour note was its preparation, found to be complex and expensive, which represents a serious obstacle to large‐scale applications. To tackle this issue, we wondered whether we could construct an efficient and cost‐effective peroxidase‐mimicking system via a simple, one‐pot assembly of hemin, peptide, DNA and/or other molecules in a CPDzyme manner but without the burden of covalent assembly.

**Figure 1:**
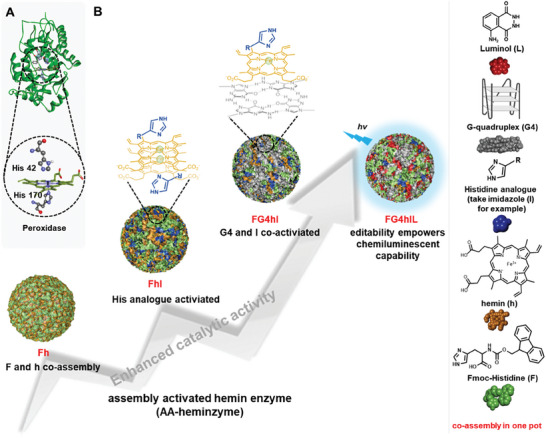
A) Key amino acid residues in the heme‐binding region of HRP. B) Schematic representation of the assembly of hemin with His analogs: for instance, if imidazole (I) is used, the corresponding complex is FhI, upon addition of G4 it becomes FG4hI, and upon addition of luminol it becomes FG4hIL. All these complexes are used as AA‐heminzymes for catalyzing peroxidase‐like reactions.

We thus proposed here the new concept of assembly‐activated hemin enzyme, or AA‐heminzyme. On the basis of the significant role of histidine (His) in both HRP and CPDzyme catalytic center, which acts as both a proximal iron ligand and a distal acid‐base catalyst (**Figure** **1**A), we first assembled Fmoc‐Histidine (F), hemin (h) and His analogs (such as imidazole (I) for example) in a complex referred to as Fmoc‐Histidine/hemin/imidazole (FhI). Then, a G4 structure was added to provide a Fmoc‐Histidine/G4/hemin/imidazole (FG4hI) complex, which forms the very heart of AA‐heminzymes. As further demonstrated below, this system exhibits several advantages when compared to HRP, including high catalytic performance, chemical robustness and recyclability, along with a lower cost. Also, AA‐heminzymes could be easily modified to achieve new functions: for instance, we show here that FG4hI could be coupled with luminol (L) to result in FG4hIL system (Figure 1B) to detect glutathione (GSH) with the same efficiency and sensitivity than commercial kits with a far lower cost (only 2%), highlighting the huge potential of our AA‐heminzyme system.

## 2 Results and Discussion

### 2.1 Synthesis and Characterization of AA‐heminzyme

AA‐heminzyme was obtained simply by mixing His analogs (to serve as proximal/distal amino acid), hemin (cofactor), G4s (to activate hemin), and luminol (if needed) via hydrophobic effect and π‐π stacking interaction of the Fmoc group (Figure 1B),^[^ ^11^ ^]^ and the preparation details are shown in supporting information. All AA‐heminzymes were solid spherical nanoparticles, as demonstrated by transmission electron microscopy (TEM) (Figure S1, Supporting Information). Dynamic light scattering (DLS) showed that the hydrodynamic diameters of Fh, FhI, FG4hI, and FG4hIL were 284 ± 14, 299 ± 8, 315 ± 22, and 327 ± 18 nm, respectively (Figure S2A–D, Supporting Information). The zeta potential was determined to assess the charges of AA‐heminzyme and the values found (between −15 to −40 mV, Figure S2E, Supporting Information) demonstrate the stability of the nanoparticles. UV–vis absorption spectroscopy showed that hemin was incorporated as both a monomer and a dimer hemin,^[^ ^12^ ^]^ that the addition of F (Fh complex) resulted in widening and redshift of the Soret band, indicating the formation of typical disorderly J‐aggregates of hemin (Figure S3A–C, Supporting Information),^[^ ^13^ ^]^ and that of I (FhI complex) triggers a charge transfer from I to hemin, confirming their coordination (Figure S3D, Supporting Information).^[^ ^14^ ^]^ The participation of different His analogs and G4 in AA‐heminzyme, and the successful synthesis of FG4hIL were confirmed by UV–vis spectra (Figures S4,S5, Supporting Information). Finally, the actual concentration of hemin‐iron within the different complexes (Fh, FhI, and FG4hI) was quantified by ICP‐MS and found to be 5.3 ± 0.05, 6.2 ± 0.05 and 4.8 ± 0.11 µm, respectively (Figure S6, Supporting Information).

### 2.2 Screening for the Best Activators

**Figure 2:**
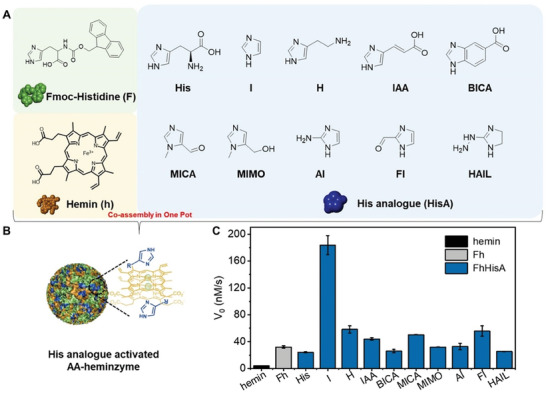
A,B) Chemical composition of Fmoc‐Histidine/hemin/histidine analogs (FhHisA) mimetic enzymes. C) The catalytic activity (H_2_O_2_‐promoted ABTS oxidation) of hemin, Fh, and FhHisA mimetic enzymes.

The activating role of His analogs within AA‐heminzymes^[^ ^15^ ^]^ was investigated in a systematic manner: nine His analogs (shown in **Figure** **2**A) were assembled within AA‐heminzymes (Figure 2B) and their catalytic activity evaluated by the model oxidation reaction of 2,2′‐azino‐bis (3‐ethylbenzothiazoline‐6‐sulfonic acid) (ABTS) in the presence of H_2_O_2_. As seen in Figure 2C, the catalytic rate of Fh alone was 32.1 nm·s^−1^, which was 7.8‐fold higher than that of free hemin. Interestingly, the addition of I, histamine (H), (2E)−3‐(1H‐Imidazole‐4‐yl) acrylic acid (IAA), 1‐Methyl‐5‐imidazole carboxaldehyde (MICA) and 2‐Formylimidazole (FI) to Fh further increased this activity, the best enhancement being obtained with I, which led to a catalytic rate of 183.7 nm·s^−1^, found to 44‐ and 5.7‐fold higher than that of hemin and Fh, respectively. It should be noted that catalytic activity of the AA‐heminzyme were affected by the ratio among different blocks: for example, when the concentration of hemin was fixed at 1 mM, the highest catalytic rate, which was 232.0 nm·s^−1^, was achieved at molar ratios of 6 and 5 for F and I, respectively (Figure S7, Supporting Information).

### 2.3 Theoretical Calculation of the Activation Mechanism

**Figure 3:**
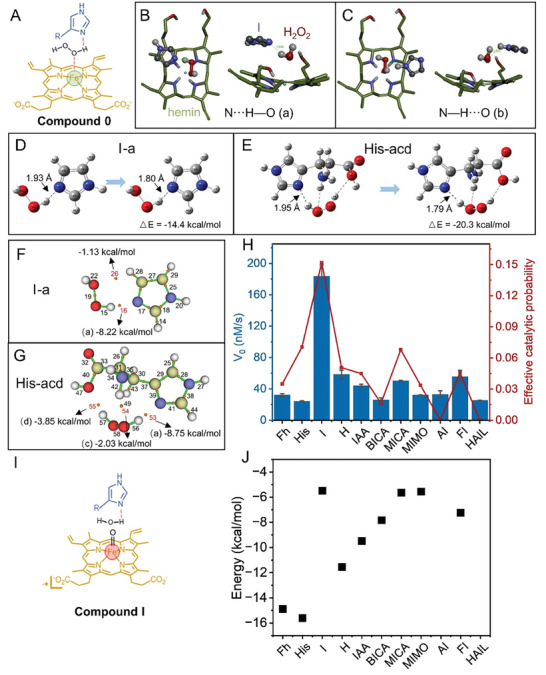
A) Schematic representation of His analog assisted H_2_O_2_ binding to hemin in compound 0. (B and C) Docking results of the H‐bond formation between I and H_2_O_2_ of either B) a‐ or C) b‐type. (D and E) Calculations of the energy of interaction between D) I, E) His and H_2_O_2_. F,G) Hydrogen bonding energy between F) I‐a, G) His‐acd, and H_2_O_2_. H) Comparison of calculated (red line) versus experimental catalytic activities (blue bar). (I) Schematic representation of compound I of His analog releasing water. J) Energy involved in breaking all the hydrogen bonds between His analogs and water during the dehydration step.

To further understand the activation role of His analogs in the catalytic systems described above, molecular docking and quantum chemical calculations were implemented. We first focused on the formation of compound 0 (**Figure** **3**A). To this end, the ability of the nitrogen atom in His analogs to act as an acid‐base catalyst when binding H_2_O_2_ in compound 0 was calculated: as seen in Table S1 (Supporting Information), a large number of predicted conformations were generated by docking (AutoDock Vina)^[^ ^16^ ^]^ with a relatively high level of spatial accuracy; all intermolecular hydrogen (H‐)bonds were classified according to whether they might facilitate the catalytic bonding process or not, following an intermolecular force analysis of all possible output conformations. For instance, N···H─O (a‐type) and N─H···O (b‐type) H‐bonds in I were artificially defined as promoting and inhibiting H‐bonds, respectively (Figure 3B,C). Notably, some molecules may generate complex situations, as shown in Figure S8 (Supporting Information), where His and H_2_O_2_ formed four states respectively His‐a, His‐ac, His‐ad, and His‐acd, which contain a‐type hydrogen bonds as well as inhibiting NH_2_···O (c‐ type) and COOH···H/O (d‐type) hydrogen bonds. We statistically analyzed the conformation of 11 molecules containing promoting (a‐type) H‐bonds (Table S1, Supporting Information) and selected the most stable conformations for further structural optimization and energy calculations with high accuracy (Gaussian software, Figure 3D,E). The energy (Δ*E*) of the interaction between these conformations and H_2_O_2_ were calculated (Table S2, Supporting Information), the H‐bonding energy predicted (Multiwfn)^[^ ^17^ ^]^ and then ranked (Figure 3F,G; Table S3, Supporting Information). The conformational frequency of effective H‐bonds (in molecular docking) and effective H‐bond energy ratio in each conformation (in Gaussian) were multiplied to obtain the effective catalytic probability (Figure 3H; Table S4, Supporting Information) which was found to be roughly consistent with the experimental results (V_0_, Figure 2C).

We then investigated the fate of compound I (Figure 3I). To this end, the ability of active N in compound I to release the produced H_2_O molecule was simulated: the H_2_O_2_ used in the previous model was replaced by H_2_O and this new model was structurally optimized (Gaussian) and the parameters used above regarding H‐bond were calculated. In addition, the binding energy required for the release of H_2_O from His analogs was also determined (Table S5, Supporting Information). The energy required to break all H‐bonds during the dehydration process was summarized in Figure 3J. For some His analogs, this process requires the breaking of not only a‐type but also other types of H‐bonds, which greatly reduced the dehydration capacity. In contrast, I, MICA, and MIMO exhibited excellent dehydration ability as no new H‐bond was formed during the dehydration step. The difference in the overall catalytic performance between I and both MICA and MIMO (Figure 3H, blue bars) thus originates in the difference in H_2_O_2_ binding (Figure 3H, red curve) as their H_2_O releasing capacity are the comparable (Figure 3J). Altogether, these in silico investigations allowed for extracting a series of criteria for evaluating the catalytic ability of H_2_O_2_/hemin‐based peroxidase‐mimicking enzymes and provided unique insights into how natural HRP avoids the formation of inhibitory H‐bonds, the donating groups in its catalytic center being involved in H‐bond with neighboring amino acids.^[^ ^10a^ ^]^

### 2.4 G‐quadruplex DNA Further Enhances the Catalytic Activity

**Figure 4:**
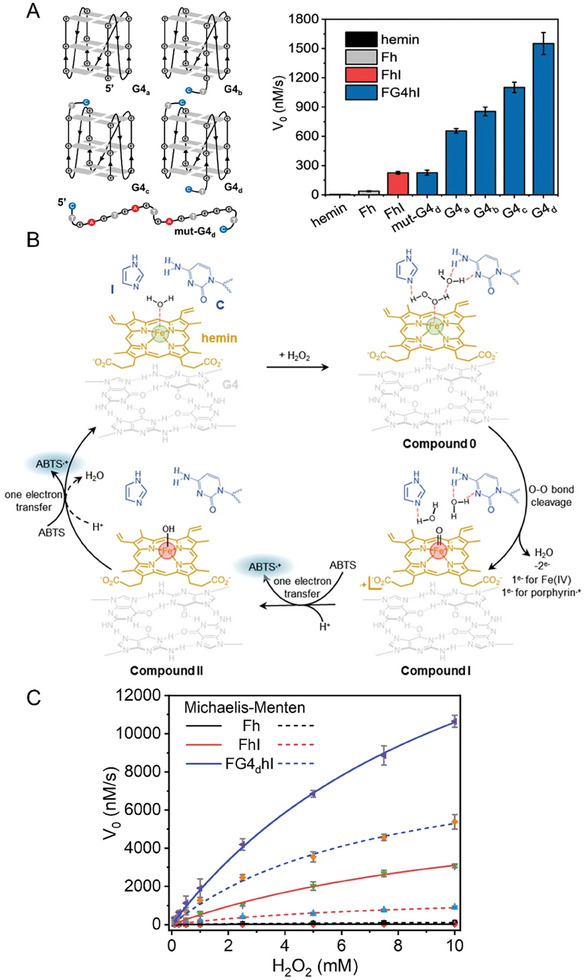
A) The effect of different G4 sequences on the catalytic activity of the corresponding AA‐heminzymes. B) Proposed catalytic cycle of FG4hI‐assisted oxidation of ABTS by H_2_O_2_. Green and red spheres represent Fe(III) and Fe(IV), respectively. C) Saturation curves of the oxidation of ABTS catalyzed by Fh, FhI and FG4_d_hI at different concentrations of H_2_O_2_. The solid and dashed lines represent the Michaelis‐Menten fitting curves at pH 5 and 7, respectively. Experiments were performed in 10 mm Britton‐Robinson buffer (pH 5 and 7, 100 mm K^+^) containing 200 nM AA‐heminzyme and 5 mm ABTS.

The results of the calculations described above indicate that I is an excellent catalytic activator, acting at both substrate binding and product releasing steps (Figure 3H,J). However, hemin dimerization, which is one of the most critical limitations of hemin‐based peroxidase‐mimicking enzymes, cannot be hampered by I. To tackle this issue, a G4 unit was used in light of its known ability to strongly interact with hemin by π–π stacking,^[^ ^3^, ^18^ ^]^ which consequently preclude its dimerization. FG4hI was thus prepared (Figure 1B) and the G4 fold of all the sequences used (Table S6, Supporting Information) was confirmed by circular dichroism (CD, Figure S9, Supporting Information). FG4hI displayed an enhanced catalytic activity (**Figure** **4**A), as demonstrated by the 2.9‐fold increment (from 225 to 655 nm·s^−1^) when parallel G4s were added, which even reaches 1551 nm·s^−1^ when using G4s with flanking dCT and dTC at both 5′ and 3′ ends,^[^ ^19^ ^]^ being thus 6.9‐ and 41.3‐fold more active than FhI and Fh, respectively. This activation effect disappeared when the sequence used was unable to fold into a G4 structure (mut‐G4). We thus report here on an unprecedented co‐activation phenomenon promoted by the concomitant action of I and G4 (Figure 4B): we propose that hemin interacts first with G4 via π–π stacking interaction; then, the distal base C of the G4 forms H‐bonds with H_2_O and helps I capture H_2_O_2_ to form compound 0, thus promoting the coordination of H_2_O_2_ to the iron center, facilitating the cleavage of the O─O bond and accelerating the formation of compound I; when encountering a first molecule of ABTS, compound I is reduced to compound II and produces ABTS·^+^; the second ABTS molecule reduces compound II to the native state of the Fe^III^ and produces another molecule of ABTS·^+^. Since the generation of compound I is the rate‐determining step of the whole catalytic reaction,^[^ ^19b^ ^]^ the co‐activation effect of I and G4 is thus critical.

### 2.5 Performance and Cost of AA‐heminzyme

To better characterize these catalytic oxidation reactions, the steady‐state kinetic curves of Fh, FhI, and FG4_d_hI were analyzed. First, the optimal pH changed from pH 7 for Fh to pH 5 for FhI and FG4_d_hI (Figure S10, Supporting Information); the kinetic parameters were thus determined at these two pH values. As shown in Figure 4C and **Table** **1**, the turnover number (*k*_cat_) values at pH 5 and 7 were 0.326 and 1.09 s^−1^ for of Fh, 35.1 and 8.23 s^−1^ for FhI, and 115 and 47.1 s^−1^ for FG4_d_hI (corresponding to an improvement > 352‐fold at pH 5, and >43‐fold at pH 7). FG4_d_hI thus reached a *k*_cat_ value which is 383‐fold higher than the common G4/Hemin system (*k*_cat_ = 0.3 s^−1^),^[^ ^20^ ^]^ that is, in the same order of magnitude than HRP (*k*_cat_ = 50–800 s^−1^).^[^ ^21^ ^]^ The *K*_m_ values also decreased (from 13.8 to 11.6 mm at pH 5; from 9.37 to 7.78 mm at pH 7, Table 1). Additionally, the *K*_m_ (ABTS) values with another substrate, ABTS, were also decreased (Figure S11, Supporting Information). These results demonstrate that the activation effect not only boosts the overall catalytic efficiency but also enhances the affinity of the enzyme for its substrates.

**Table 1:** Steady‐state kinetic parameters of Fh, FhI and FG4_d_hI at pH 5 and 7.

Catalysts	pH	*K*_m_ (mM)	*v*_max_ (µM s^−1^)	*k*_cat_ [s^−1^]	*k*_cat_/*K*_m_ [mm ^−1^ s^−1^]
Fh	5	13.8	0.0653	0.326	0.0236
	7	9.37	0.218	1.09	0.116
FhI	5	12.6	7.01	35.1	2.79
	7	8.56	1.65	8.23	0.961
FG4_d_hI	5	11.6	22.9	115	9.91
	7	7.78	9.42	47.1	6.05

To go a step further, several other factors were investigated: i) the temperature: FG4_d_hI displays an excellent resistance as its activity is 45% maintained at 95 °C for 120 min, without any change in particle size (*ca*. 330 nm at both room temperature and 95 °C) (Figure S12, Supporting Information); ii) the recyclability: FG4_d_hI is efficiently recycled, as it retains >75% of its catalytic activity after 6 recycling (centrifugation) cycles (Figure S13, Supporting Information); iii) the long‐term stability: the catalytic activity of AA‐heminzyme remained above 70% after 30 days in aqueous solution (Figure S14, Supporting Information); iv) the substrate universality: FG4_d_hI oxidizes 2 other substrates, 3,3′,5,5′‐tetramethylben‐zidine (TMB) and dopamine (DA), with an efficiency comparable to the oxidation of ABTS (Figure S15, Supporting Information); v) the catalytic cost: the catalytic cost performance, here defined as the cost required for catalyzing 1 mole of substrate at maximum catalytic efficiency, was 5.8 × 10^6^ to 2.5 × 10^5^ €/mol_sub._ (depending on the manufacturer) for HRP, 9.3 × 10^6^ €/mol_sub._ for CPDzyme, and 2.4 × 10^7^ €/mol_sub_ for G4/hemin. The catalytic cost was found to be dramatically decreased here, i.e., 2.9 × 10^2^ €/mol_sub._ for Fhl and 6.9 × 10^4^ €/mol_sub._ for FG4_d_hI, meaning that the costs of FhI and FG4_d_hI are 0.1 and 10% of that of HRP only, which makes it ideally suited to industrial applications (Tables S7–S11, Supporting Information).

### 2.6 Applications of AA‐heminzyme

**Figure 5:**
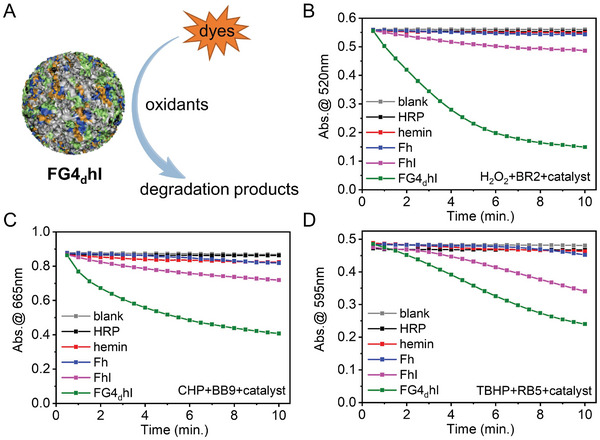
A) Schematic representation of degradation of dye using various oxidants with FG4_d_hI as a catalyst. B) Plot of maximum UV absorption versus time for BR2 decolorization by different catalysts with H_2_O_2_ as oxidant. C) Plot of maximum UV absorption versus time for BB9 decolorization by different catalysts with CHP as oxidant. D) Plot of maximum UV absorbance versus time for RB5 decolorization by different catalysts with TBHP as oxidant.

To meet the requirements of practical industrial applications, the degradation of several dye used in the textile industry was studied,^[^ ^22^ ^]^ including Basic Red 2 (BR2), N‐containing dye Basic Blue 9 (BB9) and azo dye Reactive Black 5 (RB5). These dyes are commonly used for coloring paper, bamboo and wood products as well as for dyeing acrylic, cotton, linen and silk products, and are thus common pollutants found in printing and dyeing industry wastewaters.^[^ ^23^ ^]^ Untreated wastewaters cannot be discharged into river systems as it can cause pollution and damage the ecosystem. Current physical, chemical and biological treatment strategies are effective, but with some drawbacks in terms of applicability, cost, efficacy, and time.^[^ ^24^ ^]^ Three oxidants (hydrogen peroxide, cumyl hydroperoxide and tert‐butyl hydroperoxide) and the three dyes mentioned above were selected to assess the decolorization rates catalyzed by AA‐heminzymes (Figures S16, Supporting Information). To this end, their UV absorbance was plotted against time (0 to 10 min) using different catalysts and oxidizers. As seen in **Figure** **5**, S17 (Supporting Information) (**Table** **2**), no significant changes were observed in the absence of catalyst; interestingly, in these conditions, HRP had a decolorization rate of ≈1%, that of hemin and Fh were also weak (<7%) while that of FhI reaches 12.6, 16.5, and 28.5% for BR2, BB9, and RB5, respectively, and that of FG4_d_hI was even higher (73.0, 51.5, and 50.7%, respectively). These results thus confirmed the actual application potential of AA‐heminzymes.

**Table 2:** Dye decolorization rate (%) of three oxidants by different catalysts at pH 7 within 10 min.

	blank	HRP	hemin	Fh	FhI	FG4_d_hI
H_2_O_2_‐BR2	0.359	1.11	1.25	2.59	12.6	73.0
CHP‐BB9	0.583	1.02	4.86	5.94	16.5	51.5
TBHP‐RB5	0.525	0.895	5.15	6.37	28.5	50.7

To go a step further, a luminescent substrate, luminol (L), was incorporated in FG4_d_hI, to obtain the chemiluminescent material FG4_d_hIL (Figure 1B). We aimed at using this assembly to visually detect disease‐related biomarkers: as seen in Figures S18,S19 (Supporting Information), FG4_d_hIL exhibited stronger chemiluminescence (CL) intensity than the corresponding mixture of FG4_d_hI+L over a wide range of pH (from 4 to 9). As an example, the CL intensity of FG4_d_hI+L was only 8.4% of that of FG4_d_hIL at pH 6, Figure S19 (Supporting Information), while it is found much higher when pH ≥10, likely because the luminol, whose oxidation is known to be optimized under alkaline conditions, is less accessible when embedded in the nanoparticles. We also investigated the optimal hemin: L ratio, found to be 1:6 at pH 7 (Figure S20, Supporting Information). Under these optimized conditions, we implemented the FG4_d_hIL system to detect glutathione (GSH), a well‐established biomarker of cancer^[^ ^25^ ^]^ and Alzheimer's disease.^[^ ^26^ ^]^ The efficiency of this approach relies on the H_2_O_2_‐mediated oxidation of GSH, which triggers a strong decrease of CL intensity. The optimal detection conditions for GSH were pH = 7, 50 µm H_2_O_2_ and 25 nm FG4_d_hIL (Figure S21, Supporting Information), and the limit of detection (LOD) was 0.194 nm (3σ/slope) (Figure S22A, Supporting Information). This method has a lower LOD compared to previously reported fluorescence, electrochemical and colorimetric methods (Table S12, Supporting Information). Therefore, the FG4_d_hIL‐H_2_O_2_ system could be used for the detection of GSH with good selectivity in real samples (Figure S22B, Supporting Information).

**Figure 6:**
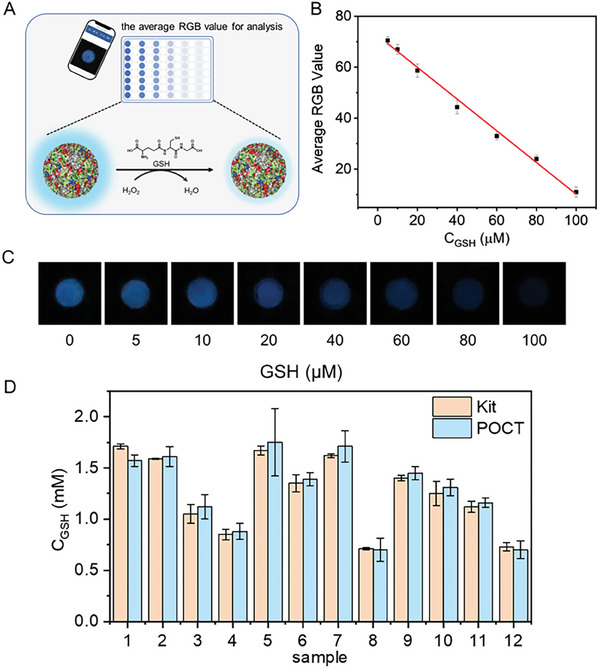
A) Schematic representation of the smartphone‐based detection of GSH by FG4_d_hIL‐H_2_O_2_. B) Calibration curve of RGB value and GSH concentration, and the linear regression equation was y = −0.62 x + 73.33 (R^2^ = 0.999). C) Chemiluminescence photographs corresponding to different GSH concentrations. FG4_d_hIL and GSH were added to 10 mM B‐R buffer (pH 7.0). The distance between the smartphone and the sample was fixed, and 100 µL H_2_O_2_ was added to trigger the CL after ensuring the surrounding environment was dark. D) Comparison of the POCT method and the kit for the detection of GSH concentration in human serum.

Finally, to further exploit this unprecedented sensitivity, we assembled a point‐of‐care GSH detection test (POCT), using a smartphone device (**Figure** **6**A): the image color was first converted into RGB values using the ColorPicker application, and the experimental points converted into a curve in order to determine a LOD, here found to be 3.33 µm (3 σ/slope) (Figure 6B). Importantly, this detection is not sensitive to the phone brand. We then used this new POCT for the detection of GSH in human serum and the results were compared with GSH Content Assay kit: as seen in Figure 6D and Table S13 (Supporting Information), the deviation rate was in the range of −8.19–6.67%, which is agreeable, for a cost decreased by *ca*. 98% (Table S14, Supporting Information), which thus confirms the great application potential of AA‐heminzymes.

## 3 Conclusion

In conclusion, we designed and assembled a peroxidase‐like mimetic enzyme referred to as AA‐heminzyme in which His analogs and G4‐forming sequences collaborate to provide hemin with an optimized environment suited to endow it with exquisite catalytic properties. The excellent performance of heminzyme was not only rationalized in silico but also demonstrated in vitro, the best prototype FG4_d_hI displaying a turnover number comparable to HRP. The ease of synthesis and the modularity of the assembly make heminzymes ideal candidates for future developments, which is further supported by both their highly catalytic efficiency and inexpensive nature. In addition, the system shows great versatility as different recognition elements (e.g., aptamers or antibodies) can be conjugated wherever needed thanks to rather simple chemical modifications, either on the hemin core (using one of its two carboxylic arms) or on the G4 part for instance (conjugating the partner of interest on one of its two ends). The first two applications reported herein, i.e., the catalytic degradation of industrial dyes (wastewater remediation) and the visual detection of glutathione biomarker (point‐of‐care testing), laid the first stones only in the construction of next‐generation artificial enzymes, whose efficiency and versatility continue to be dazzlingly inventive.

## Conflict of Interest

The authors declare no conflict of interest.

## Supporting information

Supporting Information

## Data Availability

The data that support the findings of this study are available in the supplementary material of this article.

## References

[1] a) N. A. Kotov , Science 2010, 330, 188; 20929766 10.1126/science.1190094

[2] a) K. J. Koebke , T. B. J. Pinter , W. C. Pitts , V. L. Pecoraro , Chem. Rev. 2022, 122, 12046; 35763791 10.1021/acs.chemrev.1c01025 PMC10735231

[3] a) J. L. Mergny , D. Sen , Chem. Rev. 2019, 119, 6290; 30605316 10.1021/acs.chemrev.8b00629

[4] a) P. Travascio , Y. Li , D. Sen , Chem. Biol. 1998, 5, 505; 9751647 10.1016/s1074-5521(98)90006-0

[5] a) J. Xu , R. Jiang , H. He , C. Ma , Z. Tang , Trends Anal. Chem. 2021, 139, 116257;

[6] a) T. Jian , Y. Zhou , P. Wang , W. Yang , P. Mu , X. Zhang , X. Zhang , C. L. Chen , Nat. Commun. 2022, 13, 3025; 35641490 10.1038/s41467-022-30285-9 PMC9156750

[7] Q. Liu , K. Wan , Y. Shang , Z. Wang , Y. Zhang , L. Dai , C. Wang , H. Wang , X. Shi , D. Liu , B. Ding , Nat. Mater. 2021, 20, 395. 33257794 10.1038/s41563-020-00856-6

[8] a) T. L. Poulos , Chem. Rev. 2014, 114, 3919; 24400737 10.1021/cr400415k PMC3981943

[9] a) F. Krainer , A. Glieder , Appl. Microbiol. Biotechnol. 2015, 99, 1611. 25575885 10.1007/s00253-014-6346-7 PMC4322221

[10] Y. Luo , M. Pehrsson , L. Langholm , M. Karsdal , A. Bay‐Jensen , S. Sun , Diagnostics 2023, 13, 1835. 37296687 10.3390/diagnostics13111835 PMC10252387

[11] C. Yuan , W. Ji , R. Xing , J. Li , E. Gazit , X. Yan , Nat. Rev. Chem. 2019, 3, 567. 10.1038/s41570-019-0129-8 PMC7617026 39649433

[12] J. Smalley , A. Birss , R. Withnall , J. Silver , Biochem. J. 2002, 362, 239. 11829761 10.1042/0264-6021:3620239 PMC1222381

[13] a) K. Liu , R. Xing , Y. Li , Q. Zou , H. Mohwald , X. Yan , Angew. Chem., Int. Ed. 2016, 55, 12503; 10.1002/anie.201606795 27585308

[14] a) S. Ozaki , I. Hara , T. Matsui , Y. Watanabe , Biochemistry 2001, 40, 1044; 11170427 10.1021/bi001579g

[15] a) A. Chatterjee , C. Mahato , D. Das , Angew. Chem., Int. Ed. 2021, 60, 202; 10.1002/anie.202011454 32956553

[16] a) J. Eberhardt , D. Santos‐Martins , A. F. Tillack , S. Forli , J. Chem. Inf. Model. 2021, 61, 3891; 34278794 10.1021/acs.jcim.1c00203 PMC10683950

[17] a) S. Emamian , T. Lu , H. Kruse , H. Emamian , J. Comput. Chem. 2019, 40, 2868; 31518004 10.1002/jcc.26068

[18] a) X. Mao , D. Qiu , S. Wei , X. Zhang , J. Lei , J. L. Mergny , H. Ju , J. Zhou , ACS Appl. Mater. Interfaces 2022, 14, 54598; 36459081 10.1021/acsami.2c18473

[19] a) D. Qiu , J. Mo , Y. Liu , J. Zhang , Y. Cheng , X. Zhang , Molecules 2020, 25, 3425; 32731553 10.3390/molecules25153425 PMC7435396

[20] J. Chen , J. Wang , S. C. C. van der Lubbe , M. Cheng , D. Qiu , D. Monchaud , J. L. Mergny , C. F. Guerra , H. Ju , J. Zhou , CCS Chem. 2021, 3, 2183.

[21] a) J. N. Rodriguez‐Lopez , M. A. Gilabert , J. Tudela , R. N. F. Thorneley , F. GarciaCanovas , Biochemistry 2000, 39, 13201; 11052672 10.1021/bi001150p

[22] a) S. Akhtar , A. A. Khan , Q. Husain , Chemosphere 2005, 60, 291; 15924947 10.1016/j.chemosphere.2004.12.017

[23] a) S. Payra , S. Challagulla , Y. Bobde , C. Chakraborty , B. Ghosh , S. Roy , J. Hazard. Mater. 2019, 373, 377; 30933860 10.1016/j.jhazmat.2019.03.053

[24] a) G. Crini , Bioresour. Technol. 2006, 97, 1061; 15993052 10.1016/j.biortech.2005.05.001

[25] N. Traverso , R. Ricciarelli , M. Nitti , B. Marengo , A. L. Furfaro , M. A. Pronzato , U. M. Marinari , C. Domenicotti , Oxid. Med. Cell. Longev. 2013, 2013, 972913. 23766865 10.1155/2013/972913 PMC3673338

[26] C. H. Lin , H. Y. Lane , Antioxidants 2021, 10, 1839.34829710

