# Supplementary material for: A Cost‐Effective Hemin‐Based Artificial Enzyme Allows for Practical Applications
Source: Adv Sci (Weinh). 2024 Jun 25;11(32):2402237. doi: 10.1002/advs.202402237 (PMC11348135; doi:10.1002/advs.202402237)
Supplement: Supplementary file 1 — Supporting Information [file ADVS-11-2402237-s001.docx]

Supporting Information

A Cost-Effective Hemin-based Artificial Enzyme Allows for Practical Applications

Dehui Qiu, Fangni He, Yuan Liu, Zhaoxi Zhou, Yuqin Yang, Zhongwen Long, Qianqian Chen, Desheng Chen, Shijiong Wei, Xuanxiang Mao, Xiaobo Zhang*, Jean-Louis Mergny, David Monchaud, Huangxian Ju, and Jun Zhou*

**Supporting Information Text**

**Materials and reagents**

HAP purified oligonucleotides, listed in Table S6, were purchased from Sangon Biotech (Shanghai, China) without further purification. Dimethyl sulfoxide (DMSO), hemin, hydrogen chloride (HCl), H_3_BO_3_, H_3_PO_4_, CH_3_COOH, Tris(hydroxymethyl)aminomethane (Tris), potassium chloride (KCl), potassium hydroxide (KOH), 2,2′-azino-bis (3-ethylbenzothiazoline-6-sulfonic acid) (ABTS), dopamine (DA), 3,3′,5,5′-tetramethylben-zidine (TMB), tert-Butyl hydroperoxide(TBHP), Cumyl hydroperoxide(CHP), Basic Red 2 (BR2), Basic Blue 9 (BB9), Reactive Black 5(RB5) and glutathione(GSH) were purchased from Sigma (St. Louis, USA). Hydrogen peroxide (30% H_2_O_2_) were purchased from Aladdin Ltd (Shanghai, China). Fmoc-L-Histidine (F), Histidine (His), Imidazole(I), Histamine(H), (2E)-3-(1H-Imidazole-4-yl)acrylic acid (IAA), 1H-Benzimidazole-5-carboxylic acid (BICA), 1-Methyl-5-imidazole carboxaldehyde (MICA), 1-Methyl-5-imidazole methanol (MIMO), 2-Aminoimidazole (AI), 2-Formylimidazole (FI), 2-Hydrazino-2-imidazoline (HAIL) and luminol (L) were purchased from Bide Pharmatech Ltd. Reduced Glutathione (GSH) Test Kit (DTNB colorimetric method) was purchased from Leagene Biotechnology. All other chemical reagents with analytical grade were used directly without further purification.

**Preparation and quantification of assembly-activated hemin peroxide mimetic enzyme (AA-heminzyme)**

The hemin was set at 1 mM, histidine analogs, luminol, DNA, and Fmoc-histidine stock solution were added to DMSO in a certain proportion in sequence to the total volume of 4.5 mL and sonicated for 10 min at 25°C. Then, the above DMSO solution was added to 9 mL ddH_2_O under sonicated environment, and sonication was continued for 20 min to form homogeneous nanoparticles at 25°C. Centrifugation was performed at low speed (2000 rpm, 425 rcf) for 5 min to remove large particles from the precipitate. After centrifugation at high speed (12000 rpm, 15294 rcf) for 10 min, the supernatant was discarded, and 4.5 mL ddH_2_O was added to the precipitate, which was vortexed and sonicated for 10 min to resuspend the precipitate. Repeat the above steps for three washes to remove the raw materials that did not form nanoparticles. In addition, hemin-Fe elements were measured using ICP-MS to calibrate the catalysts concentration.

**Transmission Electron Microscope (TEM) characterization of nanoparticles**

10 µL of the nanoparticle samples were added dropwise onto a 400-mesh copper mesh and dried in an oven at 60 °C before being used for TEM inspection. The samples were imaged on a JEM-2800 transmission electron microscope (JEOL Ltd, Tokyo, Japan) with a resolution of 0.16 nm, an accelerating voltage at 200 kV, and a magnification of 20k-600k. parameters such as electron beam direction, brightness, focus, and contrast were adjusted in the TEM to obtain a clear image. no staining was required for AA-heminzymes. Figure S1 was taken using a low magnification lens with a voltage of 200 kV and a magnification of 100K.

**Circular dichroism (CD) measurements**

The stock solution was diluted with 10 mM Tris-HCl (pH 7.0, 100 mM K^+^) buffer to obtain 5 μM DNA solution. CD spectra were measured with Jasco-1500 CD spectrometer in the wavelength range of 220–350 nm. The lamp was kept under a stable stream of nitrogen (99.999%) during experiments, and the measurements were repeated in triplicate at 25 °C.

**Determination of catalytic activity of AA-heminzyme.**

AA-heminzyme, ABTS were added to 10 mM Tris-HCl (pH 7.0, 100 mM K^+^) followed by H_2_O_2_. The absorbance at 420 nm (the typical UV-Vis signature of ABTS·^+^) was monitored over time with Cary100 UV-Vis spectrophotometer. The kinetic data of the first 5 s of the reaction were intercepted and fitted to obtain the initial rate of absorbance over time. The catalytic activity *V*_0_ (nM/s) was calculated by combining with the molar extinction coefficient of ABTS·^+^ at 420 nm (ε = 36,000 M^−1^·cm^−1^). Two other substrates, TMB and dopamine, were also measured: the absorbance at 652 nm (the typical UV-Vis signature of TMB·^+^, ε = 39,000 M^−1^·cm^−1^) and 480 nm (the typical UV-Vis signature of dopamine oxidation product, ε = 3,058 M^−1^·cm^−1^) as a function of absorbance over time. All kinetic measurements were repeated three times and averaged.

**Kinetic Analysis.**

The kinetics of the oxidation reactions were established by steady-state assay. The initial reaction rates were determined for different concentrations of ABTS and H_2_O_2_. The kinetic parameters were calculated according to the Michaelis-Menten equation:

$$V_{0}=\frac{V_{\max}[S]}{K_{m}+[S]}=\frac{k_{cat}{[E]}_{t}[S]}{K_{m}+[S]}$$

where *V*_0_ is the initial reaction rate, *V*_max_ is the maximum reaction rate, [S] is the concentration of substrate, *K*_m_ is the Michaelis constant, *k*_cat_ is the turnover number and [E]_t_ is the concentration of the catalyst.

**Molecular structure plotting.**

The molecular structure formulas of all molecules used in the article were drawn according to the actual structure by ChemDraw2018 software under the unified standard. After removing water molecules and other irrelevant atoms, hydrogen atoms were added to each molecular structure according to the actual situation. All molecules had their minimum energy structures optimized by the MM2 force field before importing AutodockTools (ADT ver.1.5.6) software to set torsion number and build.pdbqt file.

**Molecular docking and analysis.**

AutoDock Vina v1.2.3 software was utilized for all docking experiments to optimize the model as the docking target. The screening process was limited to molecular docking. Considering the applicability of the docking range, the docking space between the ligand molecule and H_2_O_2_ was restricted to the same side of the hemin molecule and sufficient docking space was given. The coordinates are X: -0.435, Y: -9.661, Z: 7.798 and dimensions are X: 15.0 Å, Y: 15.0 Å, Z: 7.5 Å for docking. The higher Exhaustiveness was set on 32 for relatively high accuracy. The model export amount was set to 50 to obtain a visual set of molecules containing more possible docking results for further analysis of the possible 3D conformation of each complex. For multiple ligands together with Hemin docking G4, a restricted docking space containing only the upper part of the G4 plane is used. The coordinates X: -2.768, Y: -15.976, Z: 9.536 and the dimensions X: 15.0 Å, Y: 15.0 Å, Z: 15.0 Å are used for docking. A lower Exhaustiveness value 8 was adopted in order to reduce the computational overhead. The binding affinity result file was exported as LOG for analysis finally.

**Computational Details.**

The geometrical configurations of all complexes of H_2_O_2_ and His analogs complexes were fully optimized under Gaussian16 software using the B3LYP density functional with DFT-D3(BJ) empirical dispersion correction (abbreviated as B3LYP-D3(BJ)). For the optimized geometry, the M062X-D3 method with basis group def2tzvp was evaluated by single-point energy calculations. After selecting the output fchk file, the Multiwfn 3.8 software was used to perform topology analysis for each conformation in turn, search CPs (critical points) from nuclear positions and midpoint of atomic pairs. After obtaining the relevant BCP (bond critical point) information, it was substituted into the formula to estimate its bond energy.

**Industrial dye degradation of AA-heminzyme.**

The rates of dye degradation by different oxidants were investigated using UV dynamic scanning mode (interval 30 s) in the wavelength range of 400 ~ 800 nm. H_2_O_2_-BR2, TBHP-RB5 and CHP-BB9 systems were performed with 12 μM BR2 and 1 μM catalyst were added into 10 mM Britton-Robison (B-R) buffer (pH 7.0), followed by addition of 200 μM H_2_O_2_, 500 μM TBHP and 2 mM CHP, respectively. The UV spectra were collected at 25 °C for 10 min.

**Determination of GSH by chemiluminescence (CL)**

Add 10 mM B-R buffer (pH 7.0), FG4_d_hIL and different concentrations of GSH to a 1 mL CL cuvette in a total volume of 100 μL. Then add 200 μL of H_2_O_2_ to the above solution using a flow syringe pump to trigger CL.

GSH detection by smartphone: In a 96-well plate, FG4_d_hIL and different concentrations of GSH were added to 10 mM B-R buffer (pH 7.0) with a volume of 100 μL. The distance between the phone and the 96-well plate was fixed, the camera was turned on and the video mode was selected, and 100 μL H_2_O_2_ was added to trigger the CL after ensuring the surrounding environment was dark. The CL images were captured by a video and the image color signals were converted to RGB values for analysis through ColorPicker (a mobile phone software).

**Detection of GSH in real samples**

To verify the practicality of the method, normal human serum was selected as a complex biological sample for GSH determination. Serum samples 1~4 and 5~12were obtained from Jiangsu Province Hospital and Nanjing Drum Tower Hospital, respectively. The serum was diluted 2.5 times by 10 mM PBS buffer (pH 7.0). In a 96-well plate, FG4_d_hIL and 10 μL serum dilution sample were added to 10 mM PBS buffer (pH 7.0) with a volume of 100 μL, and then 100 μL H_2_O_2_ was added to trigger the CL. The samples CL images were analyzed by the smartphone.

**Supplementary Figures**


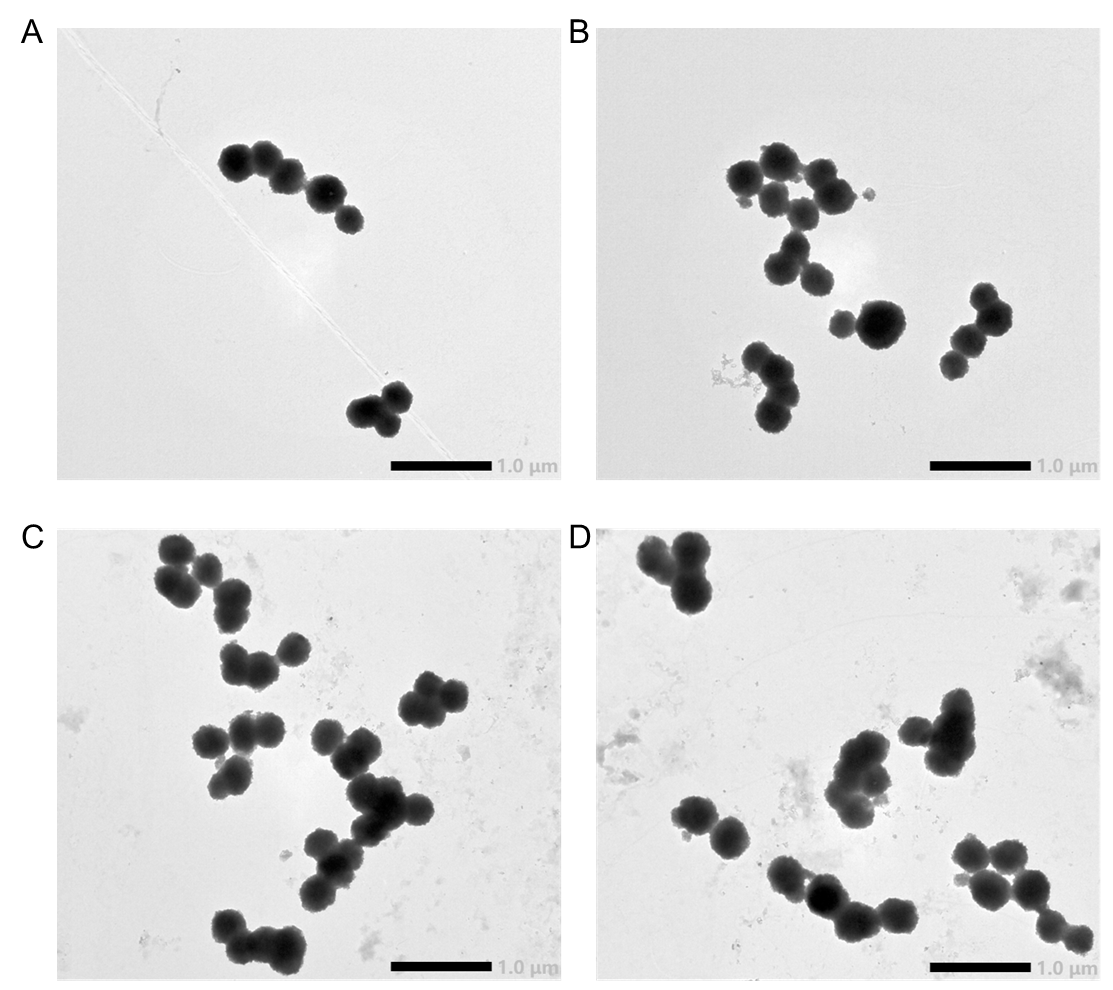


**Figure S1.** TEM images of (A) Fh, (B) FhI, (C) FG4hI and (D) FG4hIL, respectively. Acquisition information: low magnification shot, voltage is 200kV, magnification is 100K.


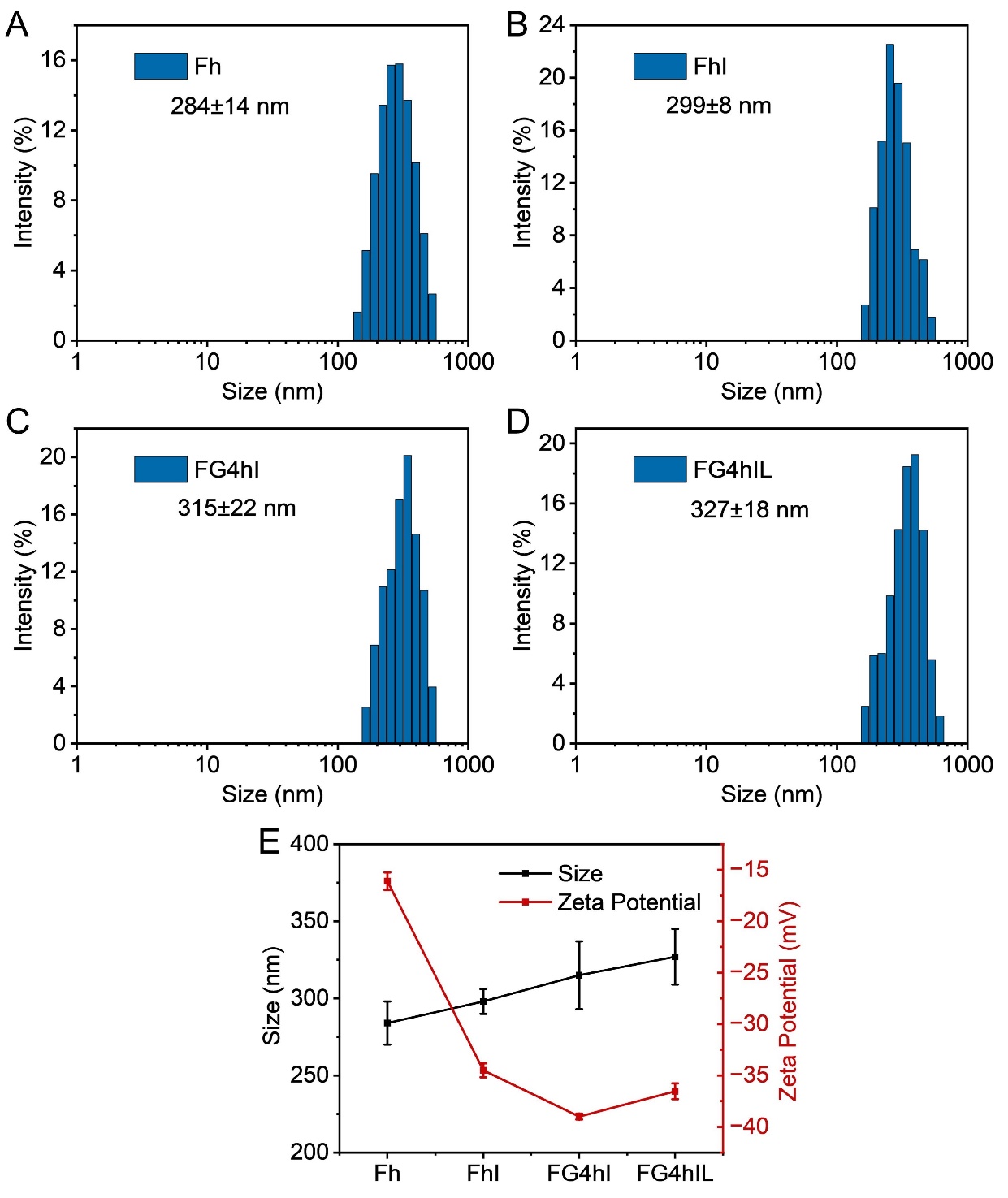


**Figure S2.** (A-D) DLS profiles of Fh, FhI, FG4hI and FG4hIL, respectively. (E) Size and zeta potential values of the samples.


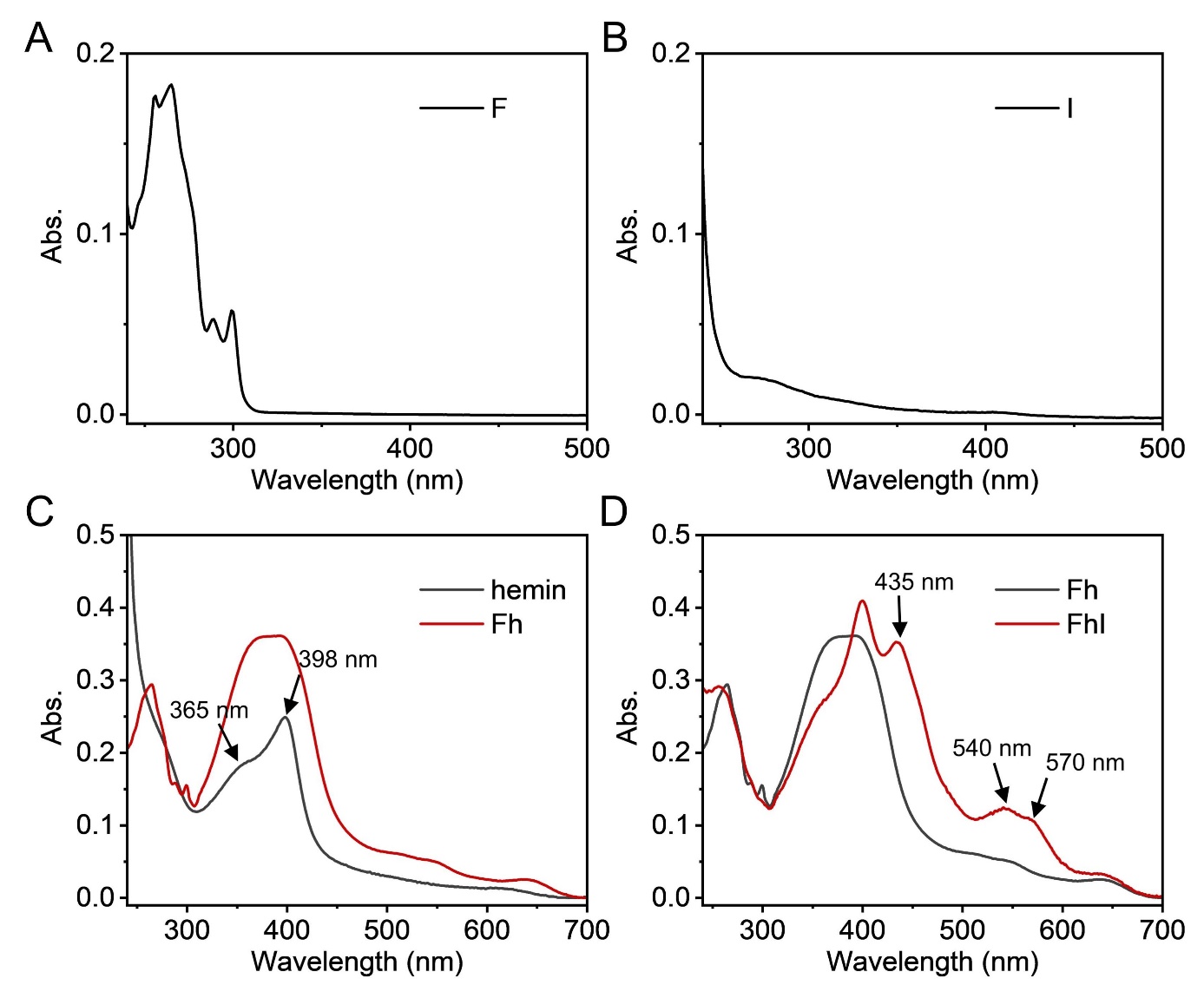


**Figure S3.** UV-Vis absorption spectra of (A) F, (B) I, (C) hemin and Fh and (D) Fh and FhI.


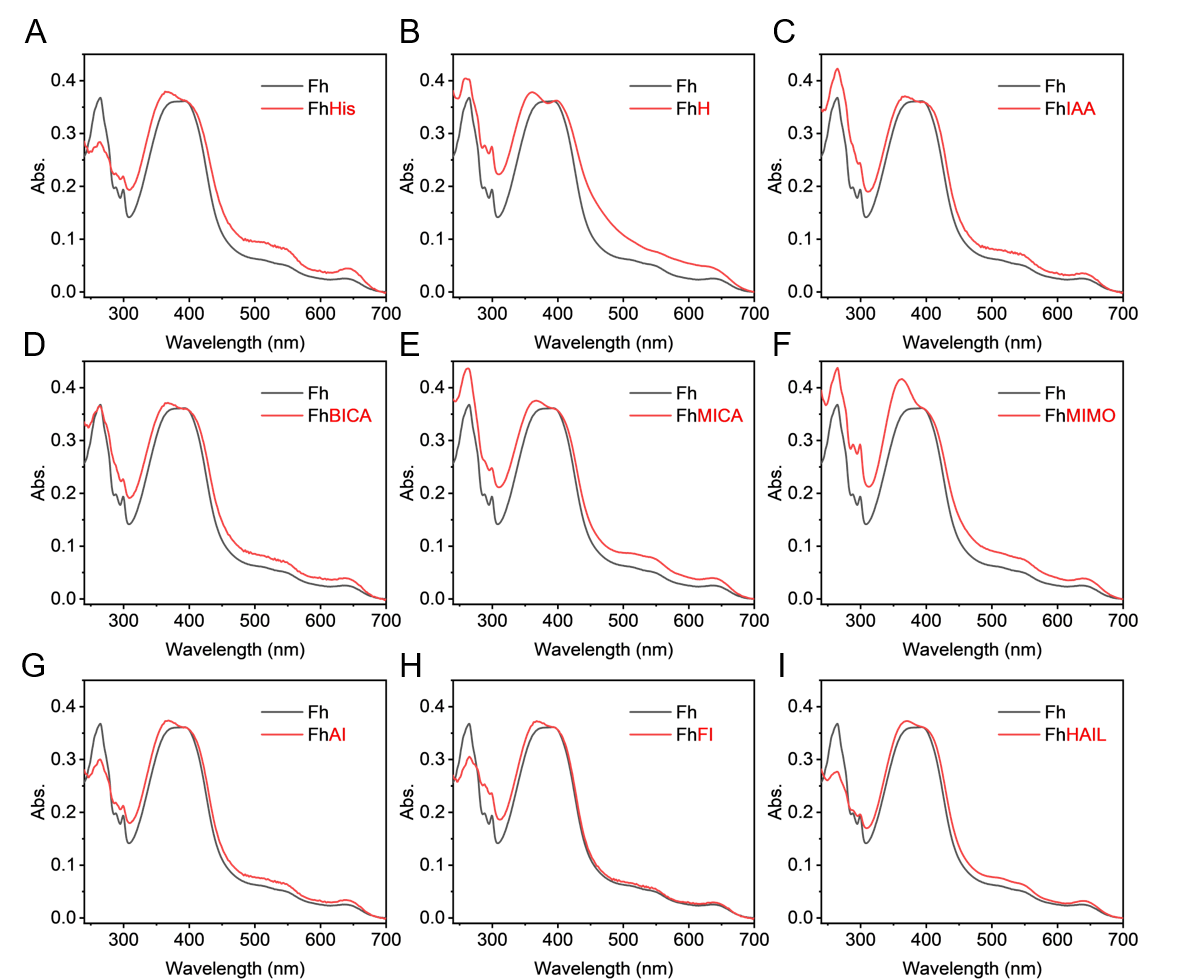


**Figure S4.** UV-Vis absorption spectra of Fh and FhHA.


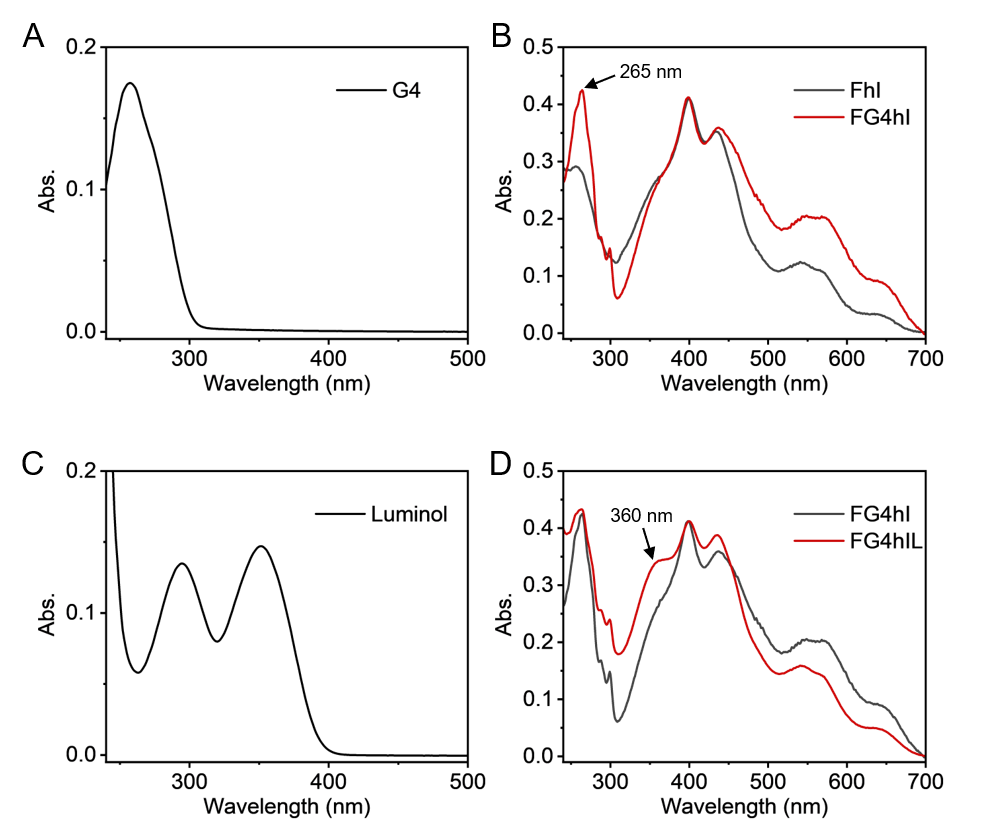


**Figure S5.** UV-Vis absorption spectra of (A) G4, (B) FhI and FG4hI, (C) Luminol and (D) FG4hI and FG4hIL.


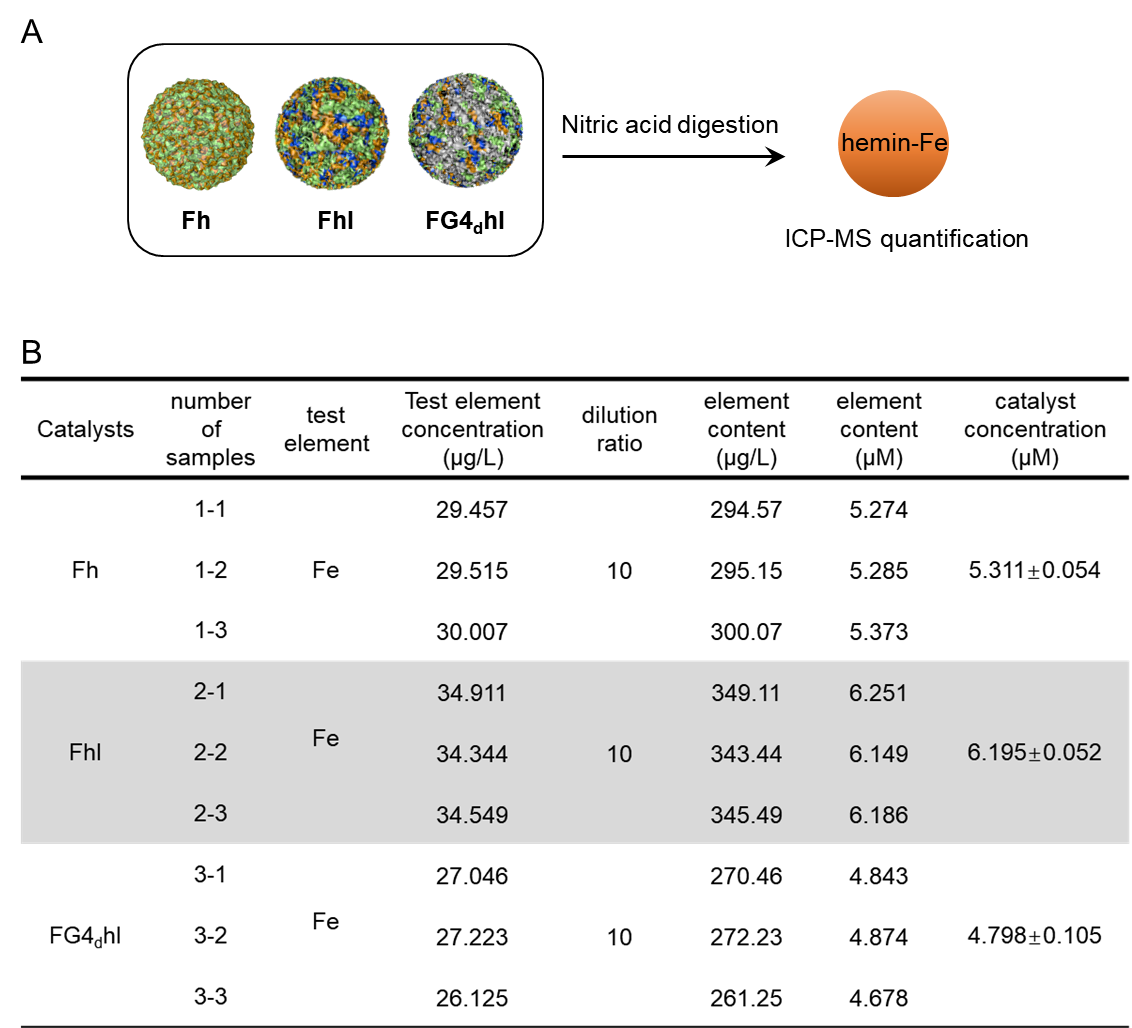


**Figure S6.** (A) Schematic and (B) data for quantification of catalysts by ICP-MS determination of hemin-Fe elemental content.


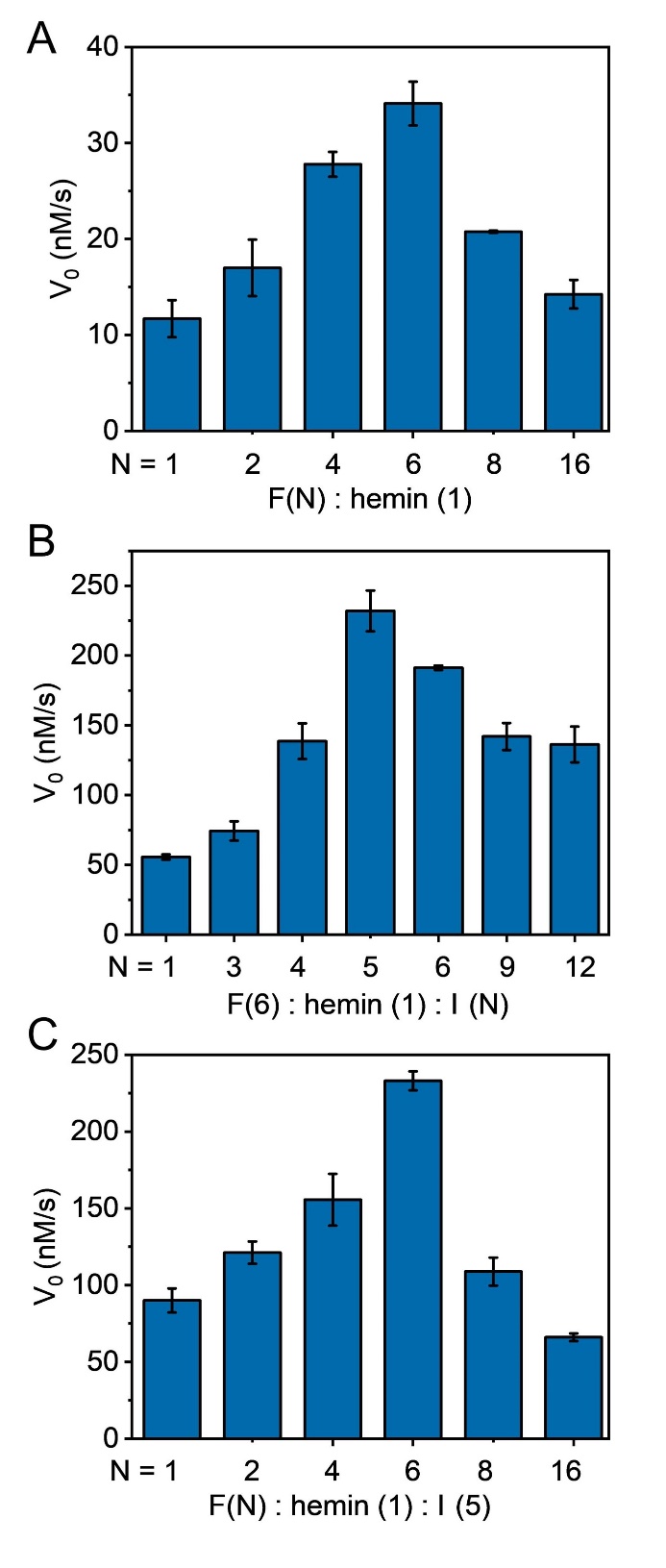


**Figure S7.** Optimization of the proportion of different blocks for AA-heminzyme synthesis. (A) The catalytic activity of Fh was obtained by adjusting the molar ratio of F to hemin. (B) The catalytic activity of FhI was obtained by adjusting the molar ratio of I when the molar ratio of F to hemin were kept 6:1. (C) The catalytic activity of FhI was obtained by adjusting the molar ratio of F when the molar ratio of hemin to I were kept 1:6.


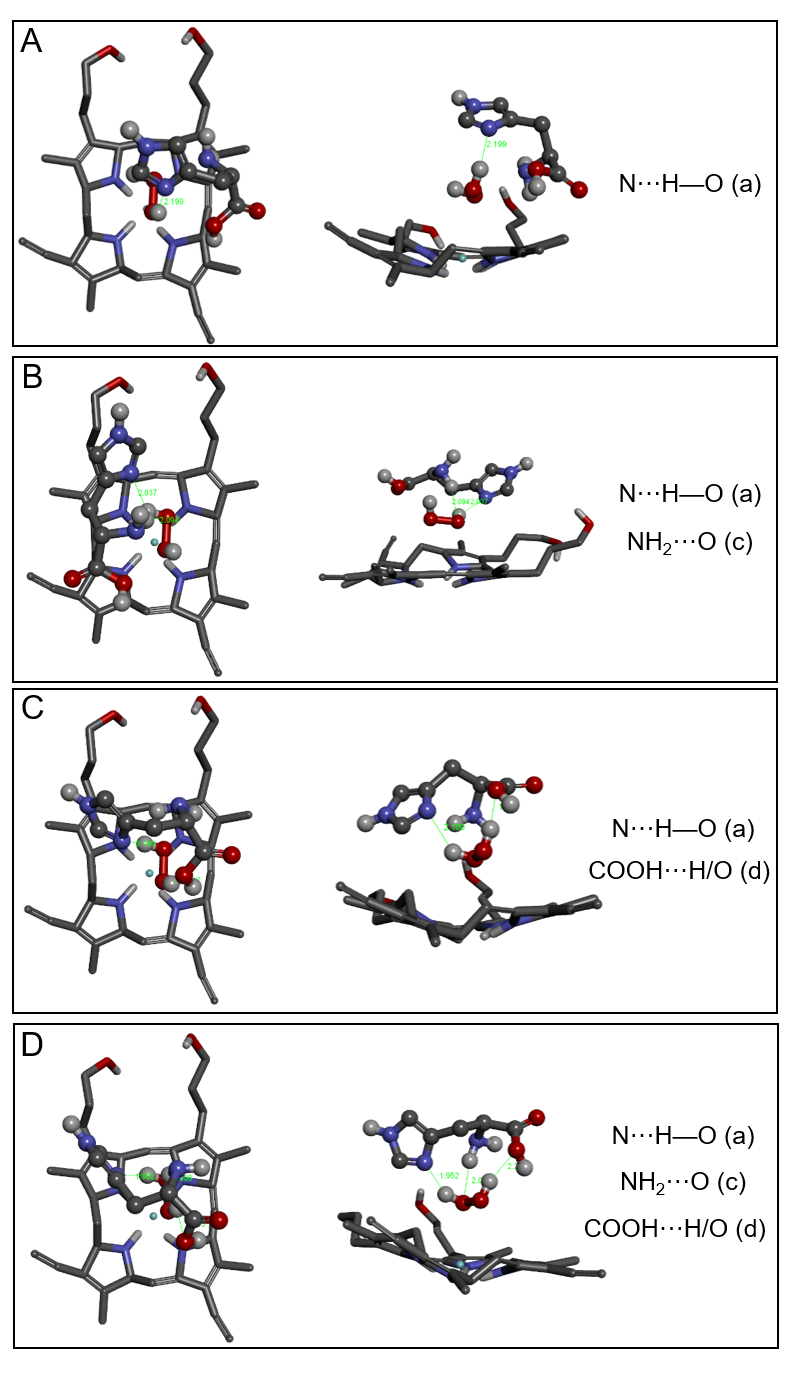
**Figure S8.** His and hemin form (A) a-type hydrogen bond, (B) a-type and c-type hydrogen bonds, (C) a-type and d-type hydrogen bonds, (D) a-type, c-type and d-type hydrogen bonds in Docking results.

**
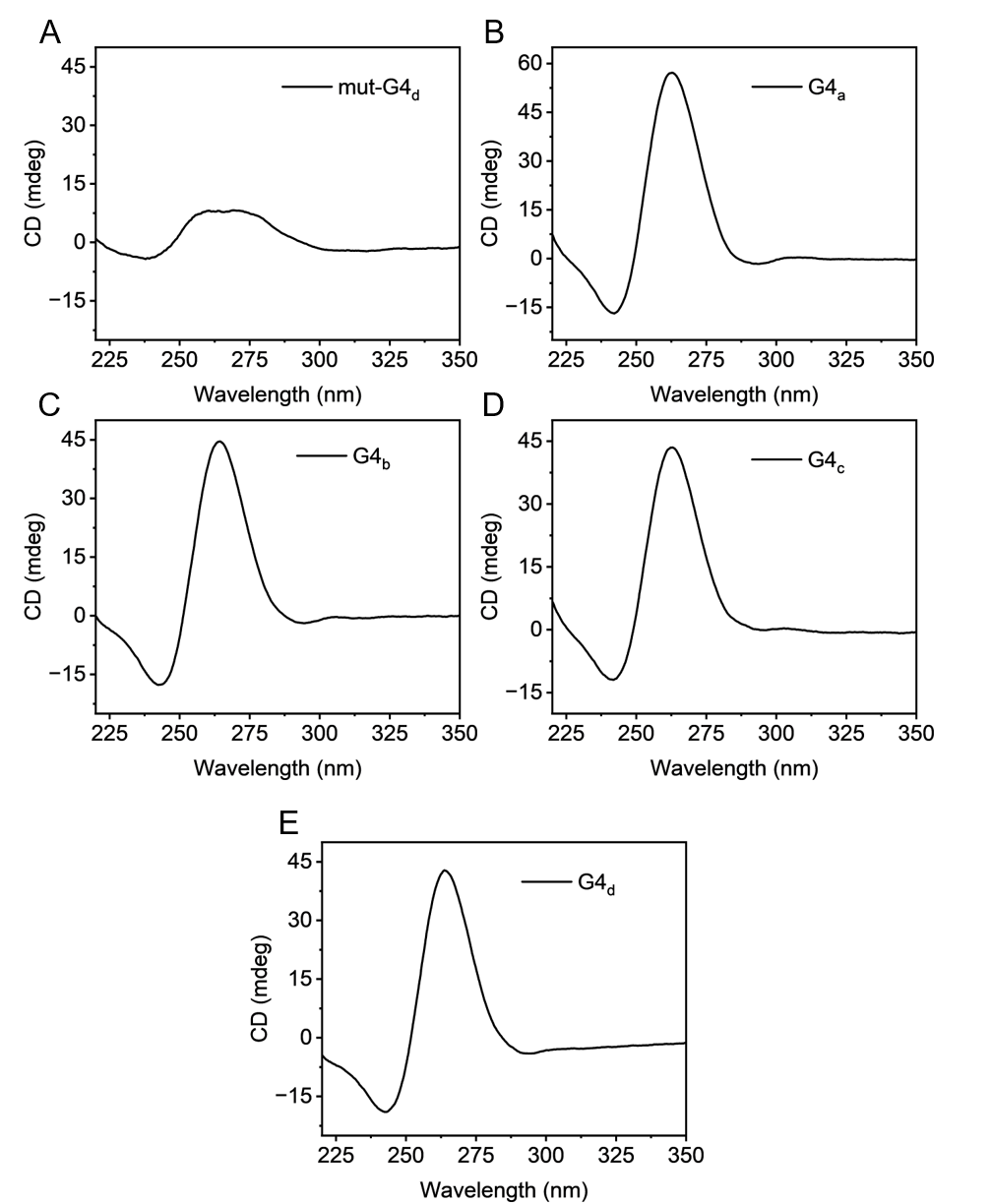
**

**Figure S9.** CD spectra of (A) mut-G4_d_, (B) G4_a_, (C) G4_b_, (D) G4_c_ and (E) G4_d_ in 10 mM Tris-HCl buffer (pH 7.0) with 100 mM KCl.

**
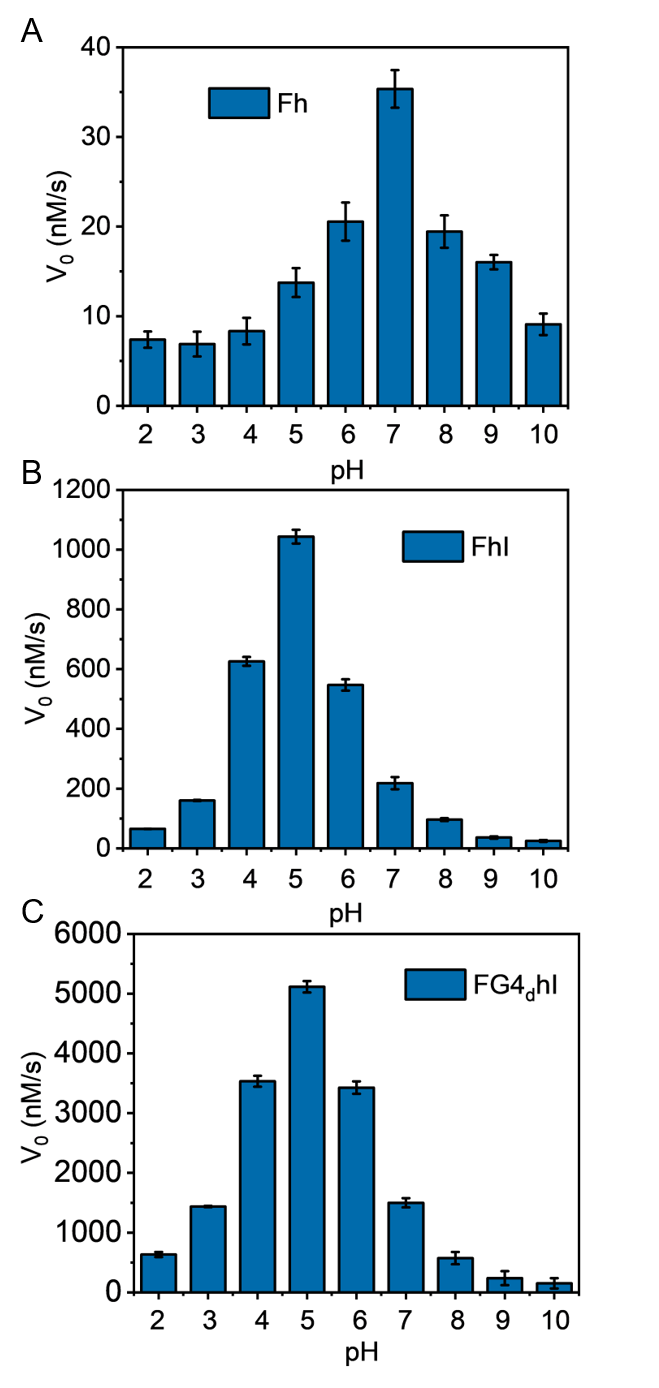
**

**Figure S10.** The catalytic activity of (A) Fh, (B) FhI and (C) FG4_d_hI for the oxidation of ABTS by H_2_O_2_ in 10 mM B-R buffer (pH 2.0 ~ 10.0) with 100 mM KCl.

**
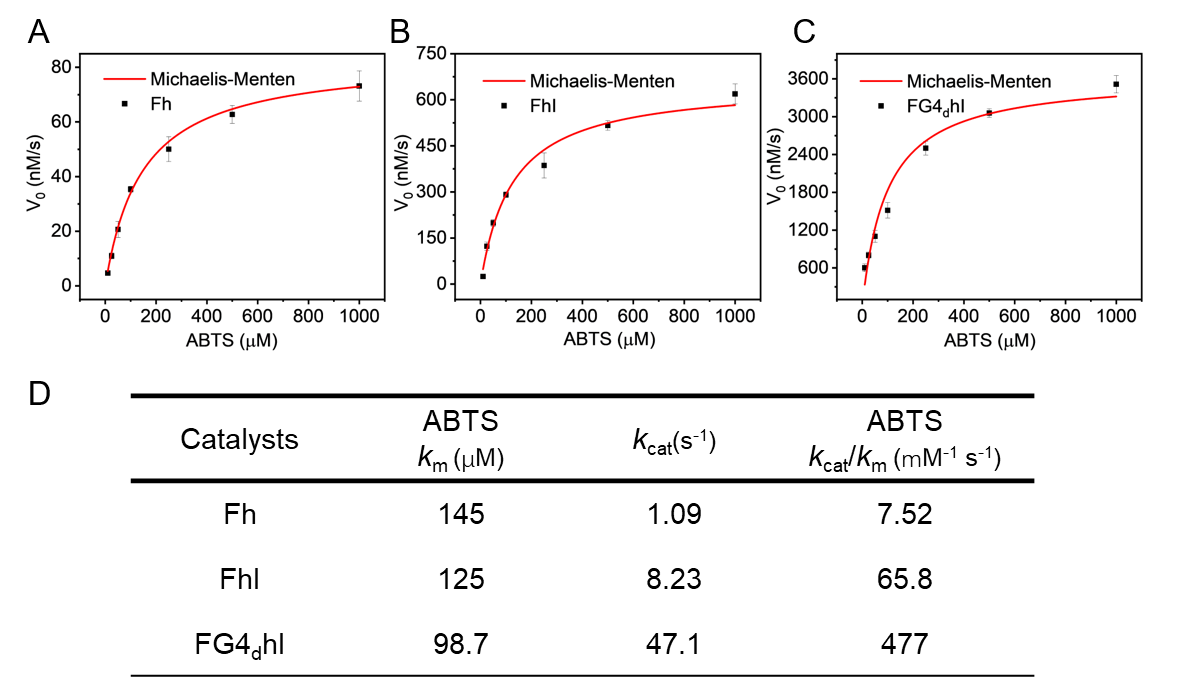
**

**Figure S11.** Saturation curves and Steady-state kinetic parameters of the oxidation of ABTS catalyzed by Fh, FhI and FG4_d_hI at different concentrations of ABTS. Experiments were performed in 10 mM B-R buffer (pH 7, 100 mM K^+^) containing 200 nM AA-heminzyme and 5 mM H_2_O_2_.

**
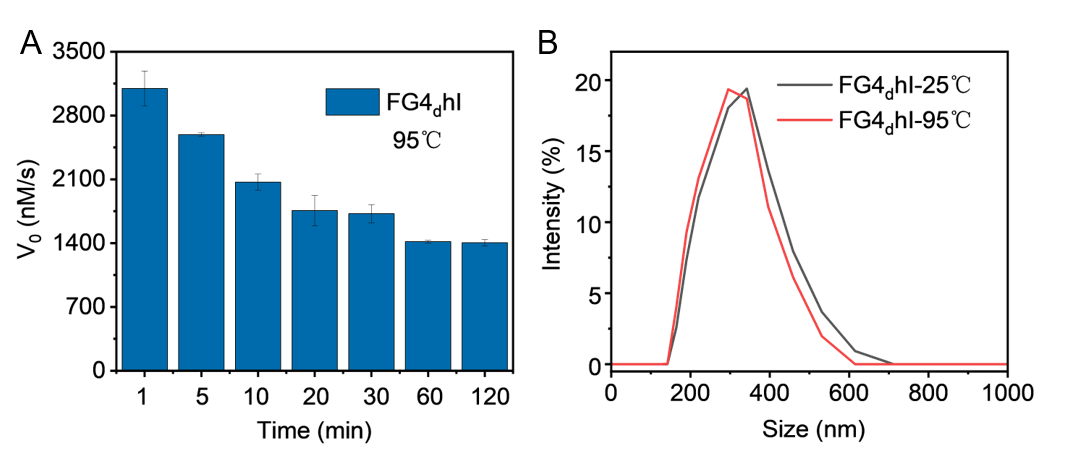
**

**Figure S12.** Characterization of thermal stability of FG4_d_hI. (A) The catalytic activity of FG4_d_hI for the oxidation of ABTS by H_2_O_2_ at 95℃ for different time. (B) The size of FG4_d_hI at 25℃ and 95℃.


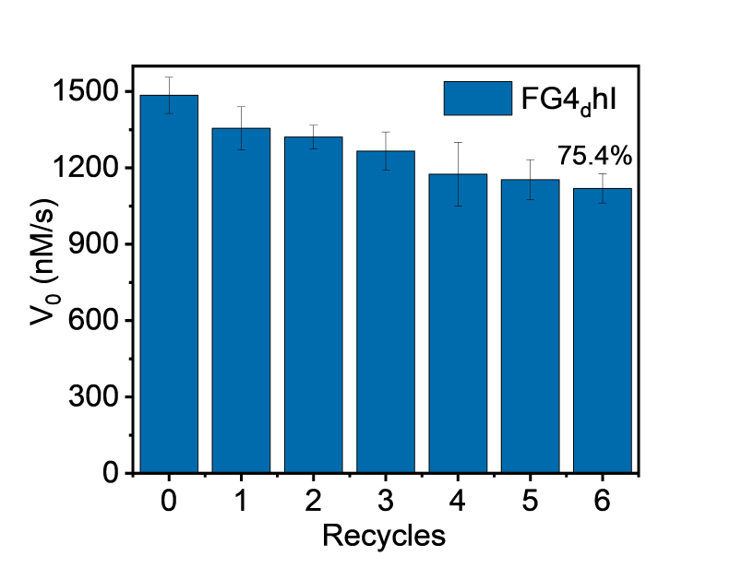


**Figure S13.** The catalytic activity of FG4_d_hI for the oxidation of ABTS by H_2_O_2_ at different recovery times.


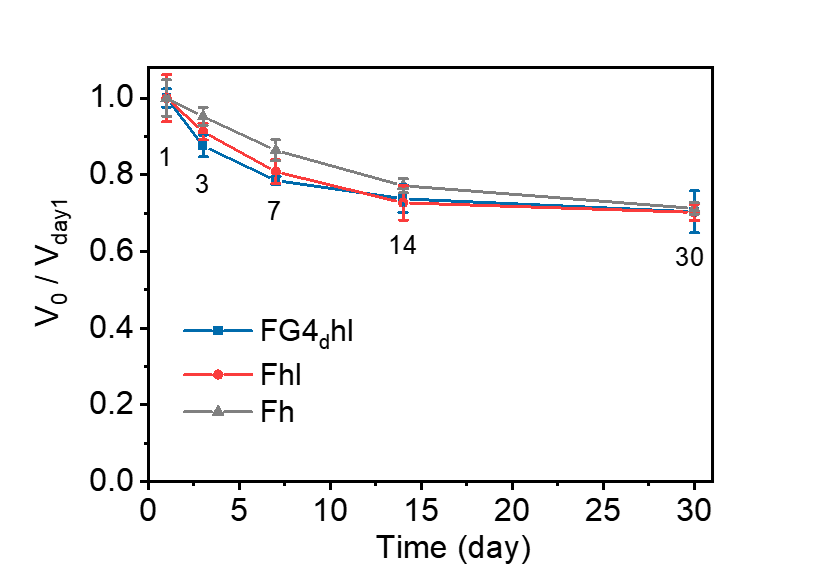


**Figure S14.** Plot of the catalytic activity of AA-heminzyme over a long period of time.


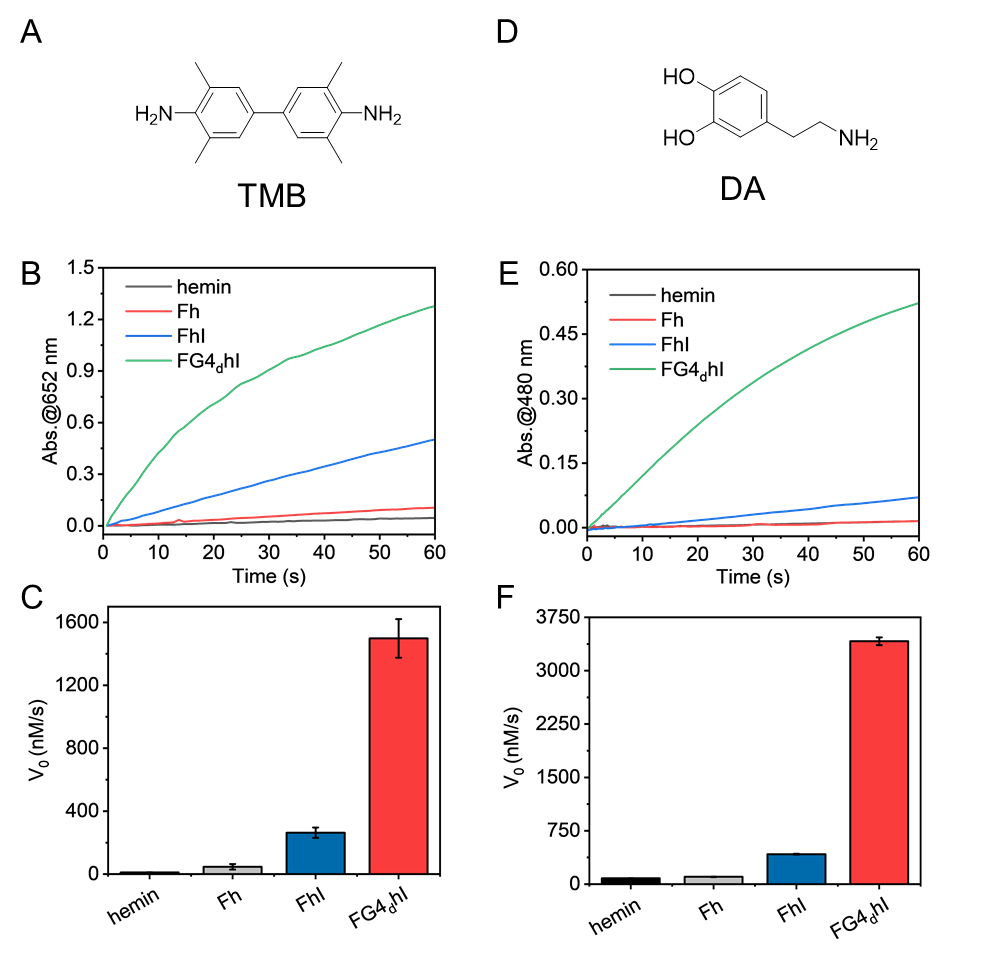


**Figure S15.** Catalytic activity measurements for different substrates with AA-heminzymes. (A) The structure formula of TMB. (B) Plots of absorbance and (C) reaction activity monitored by quantifying the absorbance signal from blue charge-transfer intermediate (TMB^·+^) at 652 nm. (D) The structure formula of DA. (E) Plots of absorbance and (F) reaction activity monitored by quantifying the absorbance signal from DA oxidation product at 480 nm.


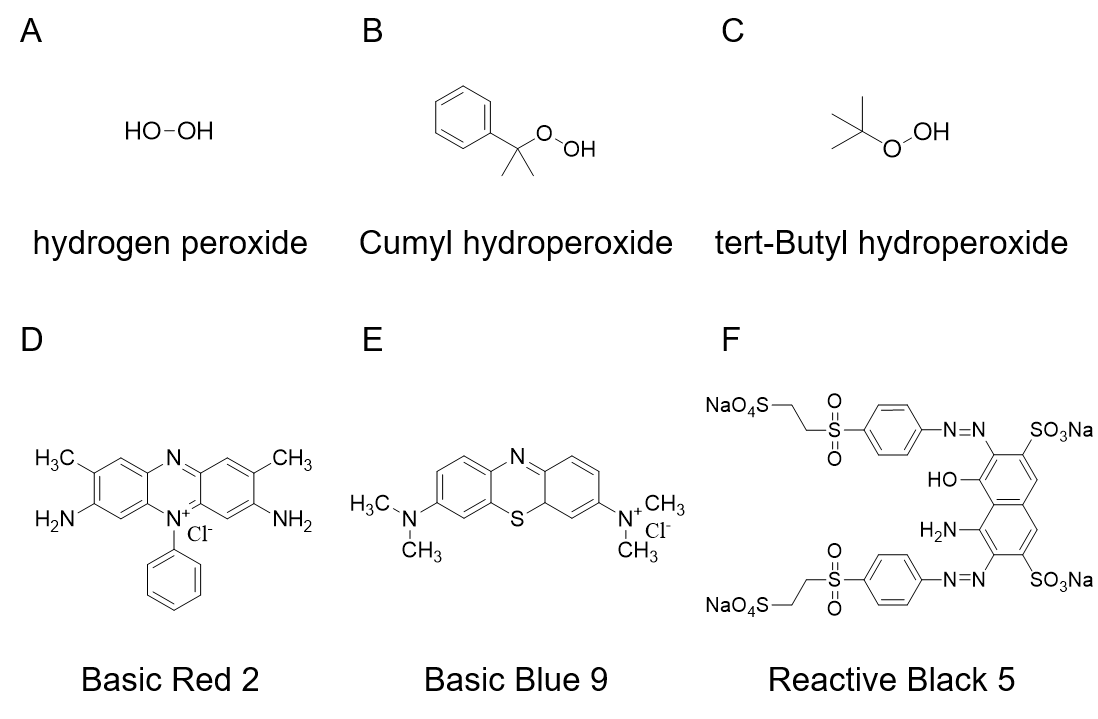


**Figure S16.** The structural formulae of the oxidants and dyes used in dye degradation.

**
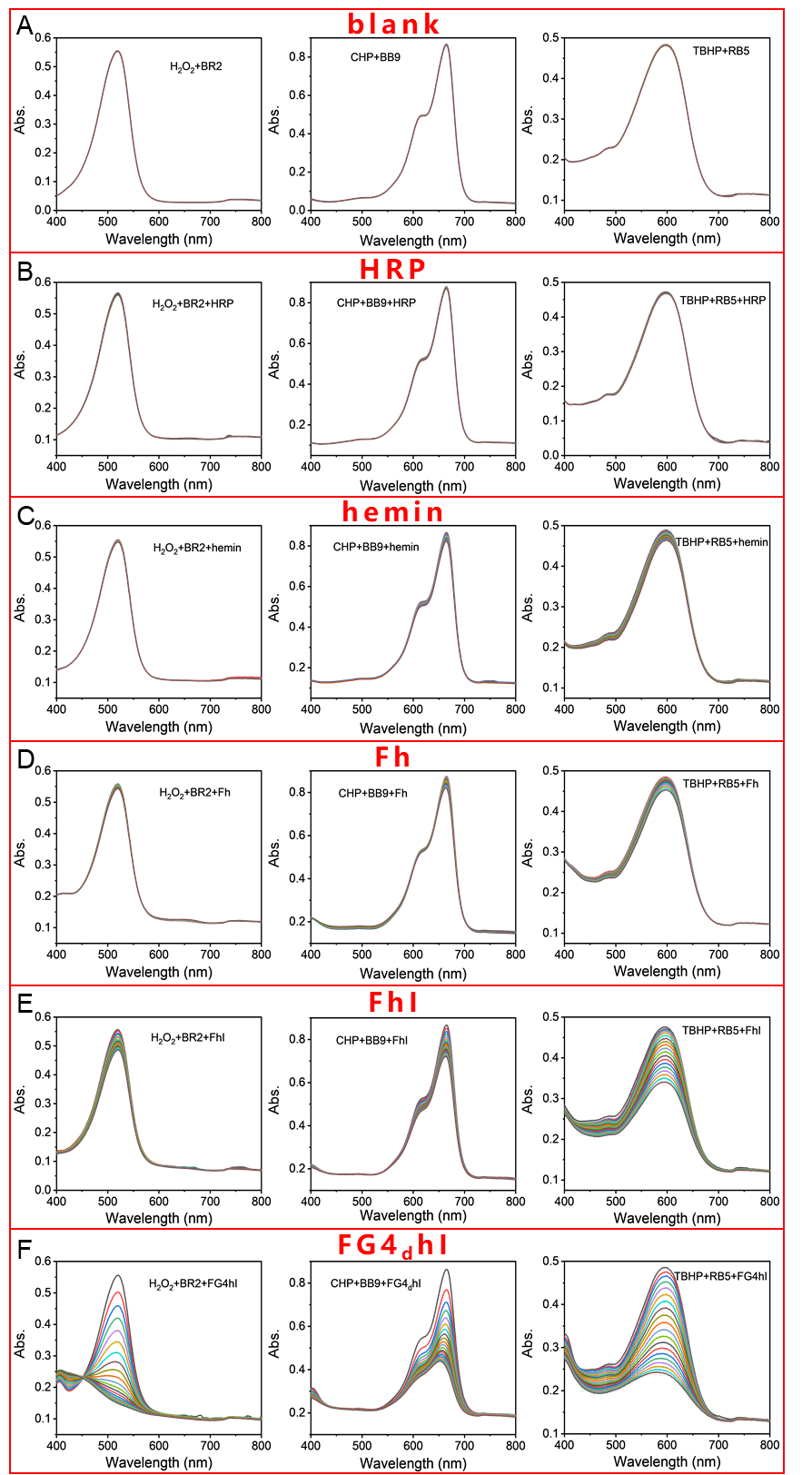
**

**Figure S17.** Dye degradation of three oxidants catalyzed by different catalysts within 10min at pH 7.


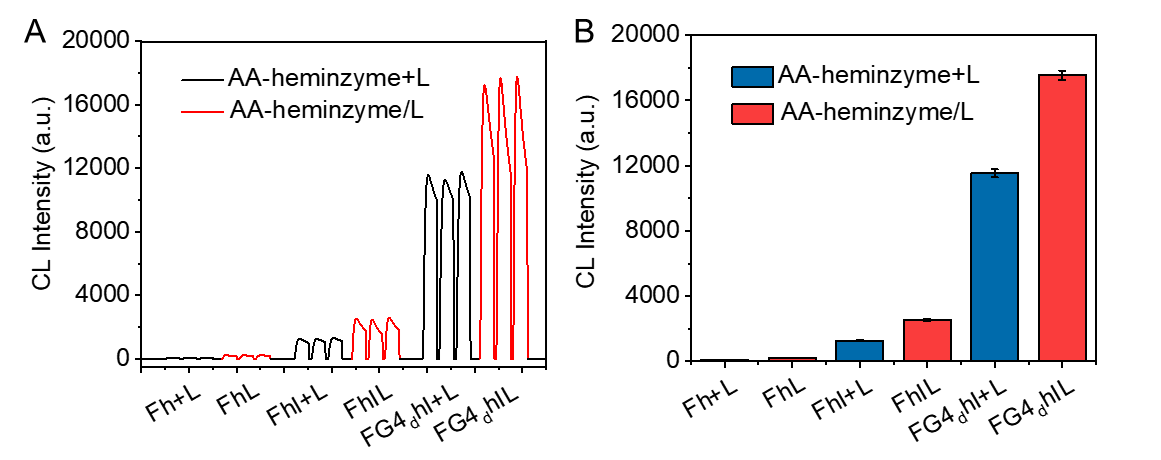


**Figure S18.** The comparison of the CL intensity of chemiluminescent material and AA-heminzyme with free luminol at pH 7.


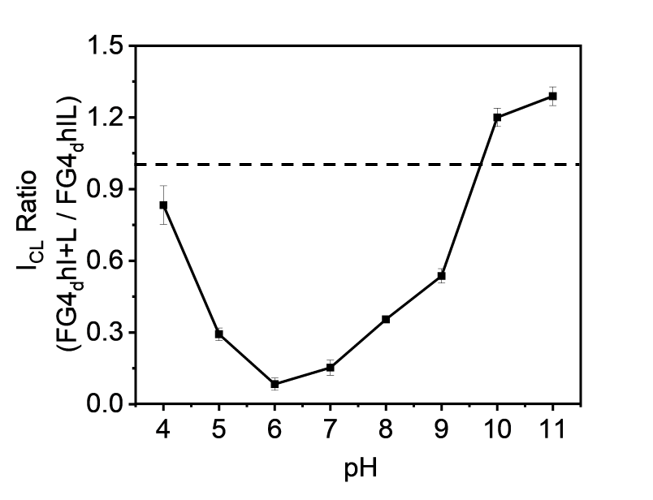


**Figure S19.** The ratio of CL intensity of FG4_d_hI + L-H_2_O_2_ system to FG4_d_hIL- H_2_O_2_ system at different pH conditions.


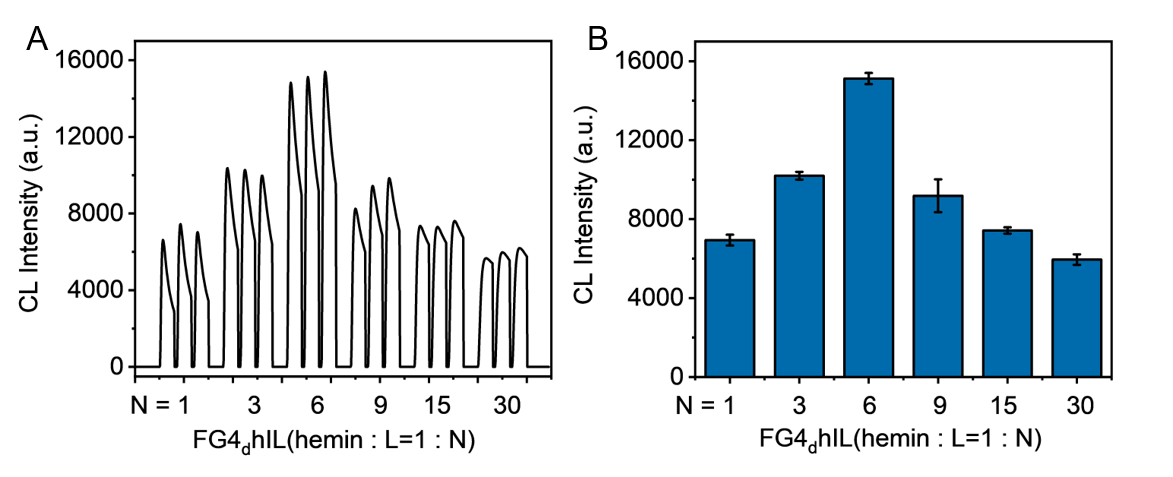


**Figure S20.** Optimization of the ratio of luminol for chemiluminescent nanomaterial synthesis. CL intensity of FG4_d_hIL doped with different ratios of luminol at pH 7.


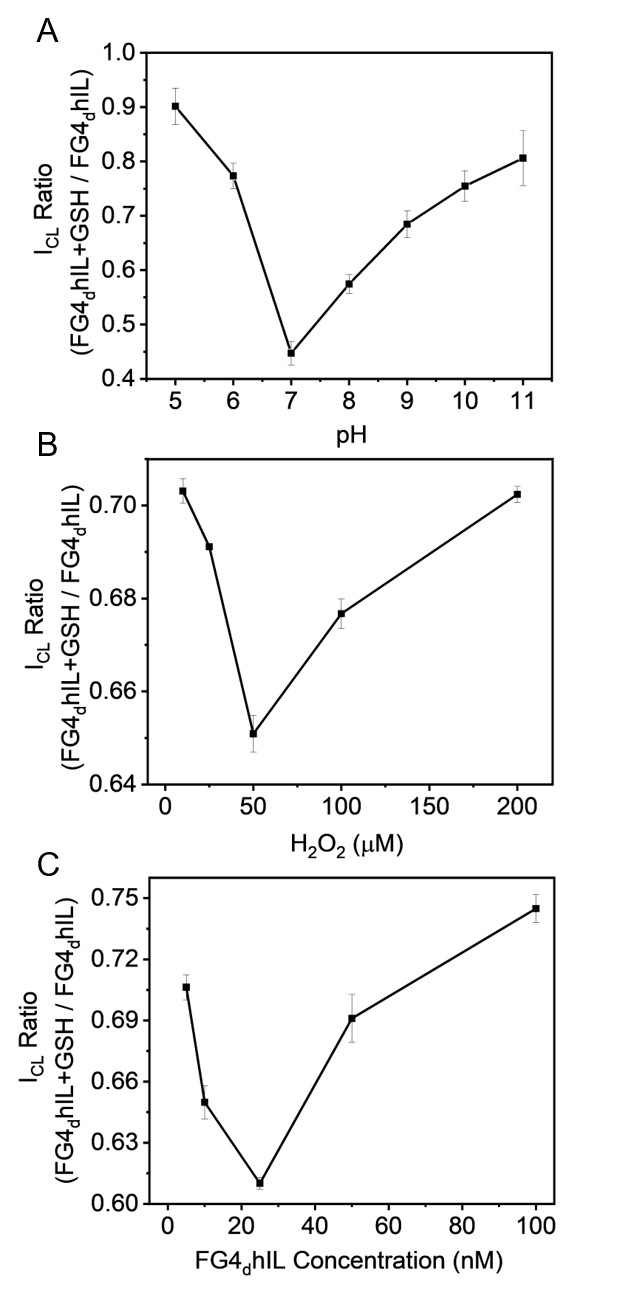
**Figure S21.** Optimization of experimental conditions for the detection of GSH with FG4_d_hIL- H_2_O_2_ system. (A) The optimization of pH. Experiments were performed in 10 mM B-R buffer (pH 5-11, 100 mM K^+^). (B) The optimization of H_2_O_2_ concentration. (C) The optimization of FG4_d_hIL concentration.


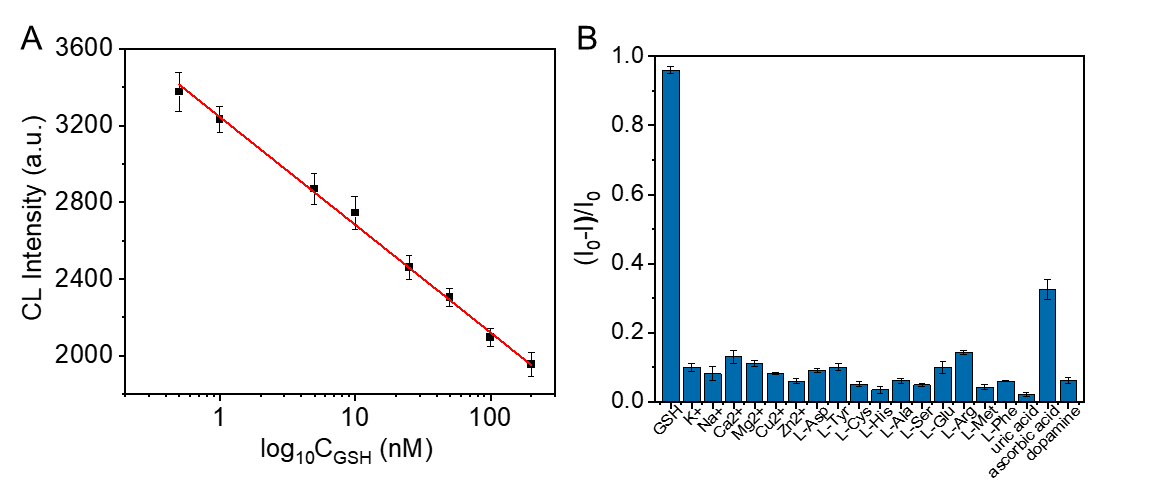


**Figure S22.** Chemiluminescent nanomaterial for GSH detection. (A) Calibration curve of CL intensity and logarithmic concentration of GSH. The linear regression equation was y = -561.99 lg x + 3244.99 (R^2^ = 0.99699) and the limit of detection (LOD) was 0.194 nM (3σ/slope). (B) (I_0_-I)/I_0_ of FG4_d_hIL-H_2_O_2_ system in presence of different interfering substances (2 μM) and GSH (200 nM).

**Supplementary Tables**

**Table S1.** Bond analysis statistics of docking results of His analogs to hemin-H_2_O_2_ system and the statistical results of different types of hydrogen bonds.

|  | **AI** |  | **BICA** |  | **F** |  | **FI** |  | **H** |  | **HAIL** |  |
| --- | --- | --- | --- | --- | --- | --- | --- | --- | --- | --- | --- | --- |
| 1 | -2.034 |  | -2.888 | d | -4.376 | d | -2.304 | d | -2.247 |  | -2.269 |  |
| 2 | -2.02 | bc | -2.847 |  | -4.356 |  | -2.303 | d | -2.169 | a | -2.264 |  |
| 3 | -1.941 | b | -2.798 |  | -4.199 | d | -2.116 |  | -2.115 |  | -2.254 |  |
| 4 | -1.933 |  | -2.775 | d | -4.189 | d | -2.079 | d | -2.079 |  | -2.226 |  |
| 5 | -1.854 | b | -2.741 |  | -4.188 | d | -2.064 | d | -2.075 |  | -2.204 |  |
| 6 | -1.853 |  | -2.726 | a | -4.157 | d | -2.052 | a | -2.071 |  | -2.172 | b |
| 7 | -1.84 | c | -2.685 |  | -4.102 |  | -2.041 | d | -2.064 |  | -2.17 |  |
| 8 | -1.821 | b | -2.624 |  | -4.058 | d | -2.041 |  | -2.055 | c | -2.167 | b |
| 9 | -1.812 | b | -2.62 |  | -3.98 | dd | -2.032 | ad | -2.034 | c | -2.157 |  |
| 10 | -1.8 |  | -2.601 |  | -3.876 |  | -2.011 | d | -2.029 |  | -2.144 |  |
| 11 | -1.782 | b | -2.543 | d | -3.844 | d | -2.008 |  | -2.026 | c | -2.122 |  |
| 12 | -1.78 | c | -2.538 |  | -3.816 |  | -2.005 |  | -2.015 |  | -2.121 |  |
| 13 | -1.779 | bc | -2.528 |  | -3.814 | cd | -1.999 | b | -2.003 |  | -2.121 |  |
| 14 | -1.778 |  | -2.517 | d | -3.814 | cd | -1.962 | d | -1.978 |  | -2.113 | b |
| 15 | -1.774 | bc | -2.511 |  | -3.762 |  | -1.959 | d | -1.955 | c | -2.108 |  |
| 16 | -1.732 |  | -2.506 |  | -3.746 | ad | -1.943 | b | -1.943 |  | -2.096 |  |
| 17 | -1.725 |  | -2.503 |  | -3.744 | a | -1.927 | d | -1.942 |  | -2.082 | b |
| 18 | -1.721 |  | -2.486 |  | -3.728 |  | -1.922 |  | -1.917 | c | -2.077 | b |
| 19 | -1.705 | c | -2.467 |  | -3.721 |  | -1.905 | d | -1.915 |  | -2.071 |  |
| 20 | -1.678 |  | -2.44 |  | -3.716 |  | -1.898 | d | -1.911 | c | -2.062 |  |
| 21 | -1.677 |  | -2.411 |  | -3.698 |  | -1.895 |  | -1.908 |  | -2.059 |  |
| 22 | -1.674 | b | -2.407 |  | -3.686 |  | -1.892 |  | -1.882 |  | -2.031 | b |
| 23 | -1.671 | bc | -2.406 | d | -3.681 | a | -1.887 |  | -1.878 | a | -2.026 |  |
| 24 | -1.66 |  | -2.401 |  | -3.666 |  | -1.881 | b | -1.845 |  | -2.025 | b |
| 25 | -1.655 | bc | -2.383 |  | -3.641 | d | -1.87 | d | -1.838 |  | -2.025 |  |
| 26 | -1.642 |  | -2.381 |  | -3.602 |  | -1.868 | d | -1.826 |  | -2.025 | b |
| 27 | -1.638 |  | -2.365 |  | -3.594 | d | -1.852 | d | -1.821 | c | -2.002 |  |
| 28 | -1.624 |  | -2.359 |  | -3.584 |  | -1.845 |  | -1.818 |  | -2.001 |  |
| 29 | -1.62 |  | -2.341 |  | -3.57 | d | -1.844 |  | -1.818 | a | -2.001 | b |
| 30 | -1.61 |  | -2.337 |  | -3.568 | dd | -1.839 |  | -1.799 | c | -1.995 |  |
| 31 | -1.607 | c | -2.332 |  | -3.541 |  | -1.816 | a | -1.72 | c | -1.994 |  |
| 32 | -1.57 |  | -2.323 |  | -3.531 | a | -1.816 |  | -1.716 | c | -1.965 |  |
| 33 | -1.568 | c | -2.32 |  | -3.503 |  | -1.788 |  | -1.715 | c | -1.955 | b |
| 34 | -1.552 |  | -2.31 |  | -3.48 | c | -1.78 |  | -1.704 |  | -1.949 |  |
| 35 | -1.549 |  | -2.31 | d | -3.477 | d | -1.767 |  | -1.694 | c | -1.929 |  |
| 36 | -1.544 |  | -2.297 | d | -3.463 | d | -1.757 |  | -1.689 | a | -1.915 |  |
| 37 | -1.544 |  | -2.253 | b | -3.46 |  | -1.738 | d | -1.683 |  | -1.915 |  |
| 38 | -1.522 |  | -2.251 |  | -3.44 |  | -1.734 |  | -1.671 |  | -1.91 | b |
| 39 | -1.507 |  | -2.242 |  | -3.435 |  | -1.731 | b | -1.667 | c | -1.898 |  |
| 40 | -1.449 |  | -2.232 |  | -3.434 | d | -1.731 |  | -1.645 |  | -1.87 |  |
| 41 | -1.399 |  | -2.22 |  | -3.43 | c | -1.713 | b | -1.639 |  | -1.84 |  |
| 42 | -1.38 |  | -2.21 |  | -3.429 |  | -1.675 |  | -1.632 |  | -1.816 |  |
| 43 | -1.377 |  | -2.203 |  | -3.421 |  | -1.651 |  | -1.631 | ac | -1.801 |  |
| 44 | -1.354 |  | -2.201 |  | -3.408 | c | -1.646 |  | -1.589 | ac | -1.797 | b |
| 45 |  |  | -2.189 |  | -3.403 | cd | -1.626 | d | -1.576 | c | -1.791 | b |
| 46 |  |  | -2.18 |  | -3.275 | c | -1.601 |  | -1.573 |  | -1.752 |  |
| 47 |  |  | -2.166 | d | -3.273 |  | -1.597 |  | -1.55 |  |  |  |
| 48 |  |  |  |  | -3.24 | d | -1.586 |  | -1.543 | c |  |  |
| 49 |  |  |  |  |  |  | -1.538 |  | -1.525 |  |  |  |
| 50 |  |  |  |  |  |  | -1.535 | b | -1.491 |  |  |  |
|  |  |  |  |  |  |  |  |  |  |  |  |  |
| **Count** | 44 |  | 47 |  | 48 |  | 50 |  | 50 |  | 46 |  |
| **All_a** |  | 0 |  | 1 |  | 4 |  | 3 |  | 6 |  | 0 |
| **a** |  | 0 |  | 1 |  | 3 |  | 2 |  | 4 |  | 0 |
| **ac** |  | 0 |  | 0 |  | 0 |  | 0 |  | 2 |  | 0 |
| **ad** |  | 0 |  | 0 |  | 1 |  | 1 |  | 0 |  | 0 |
| **acd** |  | 0 |  | 0 |  | 0 |  | 0 |  | 0 |  | 0 |
| **ab** |  | 0 |  | 0 |  | 0 |  | 0 |  | 0 |  | 0 |
| **abd** |  | 0 |  | 0 |  | 0 |  | 0 |  | 0 |  | 0 |
| **abc** |  | 0 |  | 0 |  | 0 |  | 0 |  | 0 |  | 0 |
|  | **His** |  | **IAA** |  | **I** |  | **MICA** |  | **MIMO** |  |  |  |
| 1 | -2.638 | cd | -2.381 |  | -1.647 | b | -2.059 | d | -2.004 | d |  |  |
| 2 | -2.612 | d | -2.348 |  | -1.645 | a | -2.042 | d | -1.98 |  |  |  |
| 3 | -2.569 | ac | -2.336 |  | -1.607 | a | -2.011 | d | -1.95 |  |  |  |
| 4 | -2.559 | a | -2.273 | d | -1.597 | b | -1.947 | a | -1.87 | d |  |  |
| 5 | -2.529 |  | -2.196 |  | -1.597 | b | -1.926 | d | -1.862 |  |  |  |
| 6 | -2.507 | d | -2.184 | d | -1.507 |  | -1.925 | d | -1.857 | a |  |  |
| 7 | -2.482 | ac | -2.183 |  | -1.5 |  | -1.915 | d | -1.847 | d |  |  |
| 8 | -2.422 | acd | -2.183 |  | -1.489 | b | -1.914 | d | -1.843 | d |  |  |
| 9 | -2.422 |  | -2.152 |  | -1.474 |  | -1.909 |  | -1.842 |  |  |  |
| 10 | -2.421 | d | -2.147 |  | -1.47 | b | -1.904 | a | -1.841 |  |  |  |
| 11 | -2.375 | ad | -2.143 |  | -1.459 |  | -1.894 | d | -1.835 | d |  |  |
| 12 | -2.367 |  | -2.138 |  | -1.455 |  | -1.854 | d | -1.832 | d |  |  |
| 13 | -2.349 |  | -2.134 | a | -1.435 | a | -1.819 |  | -1.82 | a |  |  |
| 14 | -2.335 | cd | -2.107 |  | -1.43 | b | -1.803 |  | -1.81 |  |  |  |
| 15 | -2.335 | c | -2.093 |  | -1.429 | a | -1.795 | d | -1.793 |  |  |  |
| 16 | -2.333 |  | -2.077 |  | -1.393 | b | -1.794 | d | -1.768 |  |  |  |
| 17 | -2.278 |  | -2.075 |  | -1.39 | a | -1.793 |  | -1.746 | d |  |  |
| 18 | -2.274 | a | -2.066 | a | -1.362 |  | -1.784 | d | -1.738 |  |  |  |
| 19 | -2.261 |  | -2.066 |  | -1.36 |  | -1.779 | d | -1.733 |  |  |  |
| 20 | -2.261 | d | -2.066 | d | -1.349 | a | -1.778 |  | -1.725 | d |  |  |
| 21 | -2.235 | d | -2.055 |  | -1.337 | b | -1.774 | d | -1.723 | d |  |  |
| 22 | -2.231 | c | -2.05 |  | -1.336 |  | -1.772 | d | -1.718 |  |  |  |
| 23 | -2.223 | ac | -2.044 |  | -1.316 |  | -1.771 | d | -1.707 |  |  |  |
| 24 | -2.213 |  | -2.021 |  | -1.303 |  | -1.767 | d | -1.701 |  |  |  |
| 25 | -2.206 | c | -2.016 |  | -1.3 |  | -1.766 |  | -1.697 |  |  |  |
| 26 | -2.199 | d | -2.013 |  | -1.299 |  | -1.765 | d | -1.687 | d |  |  |
| 27 | -2.191 |  | -1.962 | d | -1.299 |  | -1.761 | d | -1.684 |  |  |  |
| 28 | -2.188 | d | -1.954 |  | -1.297 |  | -1.758 |  | -1.683 |  |  |  |
| 29 | -2.181 | d | -1.953 |  | -1.293 | b | -1.748 |  | -1.681 |  |  |  |
| 30 | -2.18 | cd | -1.94 |  | -1.265 |  | -1.731 |  | -1.666 |  |  |  |
| 31 | -2.176 |  | -1.919 | a | -1.258 | b | -1.719 |  | -1.664 |  |  |  |
| 32 | -2.14 | cd | -1.889 |  | -1.256 | a | -1.717 |  | -1.661 |  |  |  |
| 33 | -2.124 | d | -1.843 |  | -1.254 | a | -1.711 |  | -1.647 |  |  |  |
| 34 | -2.1 |  | -1.826 | a | -1.249 | b | -1.706 | d | -1.641 |  |  |  |
| 35 | -2.088 | d | -1.802 |  | -1.244 |  | -1.705 |  | -1.64 |  |  |  |
| 36 | -2.066 |  | -1.796 |  | -1.239 |  | -1.702 |  | -1.626 | d |  |  |
| 37 | -2.062 |  | -1.782 |  | -1.238 |  | -1.7 |  | -1.593 | d |  |  |
| 38 | -2.039 |  | -1.782 | a | -1.18 |  | -1.693 | a | -1.592 |  |  |  |
| 39 | -2.025 | d | -1.753 |  | -1.174 |  | -1.688 | d | -1.587 | d |  |  |
| 40 | -2.021 | d | -1.745 |  | -1.167 |  | -1.688 | d | -1.582 |  |  |  |
| 41 | -2.019 |  | -1.744 |  | -1.15 |  | -1.681 |  | -1.57 | d |  |  |
| 42 | -1.983 | cd | -1.733 |  | -1.104 |  | -1.653 | d | -1.568 |  |  |  |
| 43 | -1.979 | d | -1.685 | a | -1.088 |  | -1.639 | a | -1.558 | d |  |  |
| 44 | -1.964 |  | -1.672 |  | -1.08 |  | -1.632 | d | -1.554 |  |  |  |
| 45 | -1.962 |  | -1.669 | d | -1.061 |  | -1.625 |  | -1.541 | d |  |  |
| 46 | -1.961 |  | -1.666 |  | -1.038 |  | -1.621 | d | -1.537 |  |  |  |
| 47 | -1.953 | d | -1.632 |  | -0.976 | a | -1.613 |  | -1.525 |  |  |  |
| 48 | -1.939 | c | -1.631 |  |  |  | -1.557 |  | -1.465 | d |  |  |
| 49 | -1.91 |  | -0.767 |  |  |  | -1.551 |  |  |  |  |  |
| 50 |  |  |  |  |  |  |  |  |  |  |  |  |
|  |  |  |  |  |  |  |  |  |  |  |  |  |
| **Count** | 49 |  | 49 |  | 47 |  | 49 |  | 48 |  |  |  |
| **All_a** |  | 7 |  | 6 |  | 9 |  | 4 |  | 2 |  |  |
| **a** |  | 2 |  | 6 |  | 9 |  | 4 |  | 2 |  |  |
| **ac** |  | 3 |  | 0 |  | 0 |  | 0 |  | 0 |  |  |
| **ad** |  | 1 |  | 0 |  | 0 |  | 0 |  | 0 |  |  |
| **acd** |  | 1 |  | 0 |  | 0 |  | 0 |  | 0 |  |  |
| **ab** |  | 0 |  | 0 |  | 0 |  | 0 |  | 0 |  |  |
| **abd** |  | 0 |  | 0 |  | 0 |  | 0 |  | 0 |  |  |
| **abc** |  | 0 |  | 0 |  | 0 |  | 0 |  | 0 |  |  |

**Table S2.** The intermolecular force between His analogs and H_2_O_2_ obtained by subtracting the energy of dislodging H_2_O_2_ from containing H_2_O_2_ after optimizing the molecular structure.

|  |  | **Dis_H_2_O_2_** | **Con_H_2_O_2_** | **H_2_O_2_** | **Δ*E* (hartree）** | **Δ*E* (kcal/mol)** |
| --- | --- | --- | --- | --- | --- | --- |
| 1 | AI_2_bc | -281.3891171 | -432.8310684 | -151.4235802 | -0.018371 | -11.528 |
| 2 | AI_3_b | -281.3891174 | -432.8398396 |  | -0.027142 | -17.032 |
| 3 | AI_7_c | -281.3891168 | -432.8295151 |  | -0.016818 | -10.554 |
| 4 | BICA_1_d | -568.0610884 | -719.5068907 |  | -0.022222 | -13.945 |
| 5 | BICA_4_d | -568.0610885 | -719.5063259 |  | -0.021657 | -13.590 |
| 6 | BICA_6_a | -568.0706663 | -719.5171824 |  | -0.022936 | -14.393 |
| 7 | BICA_37_b | -568.0711917 | -719.5120365 |  | -0.017265 | -10.834 |
| 8 | FH_1_d | -1276.06439 | -1427.519308 |  | -0.031338 | -19.665 |
| 9 | FH_8_dd | -1276.07181 | -1427.522322 |  | -0.026931 | -16.900 |
| 10 | FH_11_d | -1276.072909 | -1427.523979 |  | -0.027490 | -17.250 |
| 11 | FH_14_cd | -1276.072916 | -1427.532901 |  | -0.036404 | -22.844 |
| 12 | FH_16_ad | -1276.08847 | -1427.526858 |  | -0.014808 | -9.2919 |
| 13 | FH_17_a | -1276.08138 | -1427.537502 |  | -0.032541 | -20.420 |
| 14 | FI_1_d | -339.3069638 | -490.7546751 |  | -0.024131 | -15.143 |
| 15 | FI_4_a | -339.3069632 | -490.7525332 |  | -0.021990 | -13.799 |
| 16 | FI_6_ad | -339.3069636 | -490.754675 |  | -0.024131 | -15.143 |
| 17 | FI_13_bd | -339.3173399 | -490.7710453 |  | -0.030125 | -18.904 |
| 18 | FI_14_b | -339.31734 | -490.7703836 |  | -0.029463 | -18.489 |
| 19 | H_3_a | -359.9571778 | -511.4216369 |  | -0.040879 | -25.652 |
| 20 | H_5_c | -359.9637492 | -511.4146073 |  | -0.027278 | -17.117 |
| 21 | H_7_ac | -359.9637492 | -511.4216368 |  | -0.034308 | -21.528 |
| 22 | H_9_b | -359.9626498 | -511.4108686 |  | -0.024639 | -15.461 |
| 23 | H_11_a | -359.9626496 | -511.4198502 |  | -0.033620 | -21.097 |
| 24 | H_13_c | -359.9637491 | -511.4151643 |  | -0.027835 | -17.467 |
| 25 | HAIL_6_b | -337.8697379 | -489.3172777 |  | -0.023960 | -15.035 |
| 26 | HAIL_8_b | -337.8673685 | -489.3169051 |  | -0.025956 | -16.288 |
| 27 | HAIL_13_c | -337.8688255 | -489.3294107 |  | -0.037005 | -23.221 |
| 28 | His_1_cd | -548.4091029 | -699.8597349 |  | -0.027052 | -16.975 |
| 29 | His_3_ac | -548.4035621 | -699.8597514 |  | -0.032609 | -20.463 |
| 30 | His_4_a | -548.4003603 | -699.8634358 |  | -0.039495 | -24.784 |
| 31 | His_7_ac | -548.396731 | -699.8633521 |  | -0.043041 | -27.009 |
| 32 | His_8_acd | -548.4035622 | -699.8595502 |  | -0.032408 | -20.336 |
| 33 | His_10_d | -548.3925746 | -699.8482798 |  | -0.032125 | -20.159 |
| 34 | His_11_ad | -548.4063294 | -699.8637993 |  | -0.033890 | -21.266 |
| 35 | His_18_a | -548.405911 | -699.859754 |  | -0.030263 | -18.990 |
| 36 | IAA_4_d | -491.8702706 | -643.3115454 |  | -0.017695 | -11.104 |
| 37 | IAA_8_a | -491.8702707 | -643.3186781 |  | -0.024827 | -15.579 |
| 38 | IAA_10_a | -491.8689149 | -643.3178849 |  | -0.025390 | -15.932 |
| 39 | IAA_31_d | -491.8635176 | -643.3099532 |  | -0.022855 | -14.342 |
| 40 | I_1_a | -226.0657526 | -377.5122663 |  | -0.022934 | -14.391 |
| 41 | I_2_b | -226.0657526 | -377.5035792 |  | -0.014246 | -8.9398 |
| 42 | I_3_a | -226.0657525 | -377.5135712 |  | -0.024239 | -15.210 |
| 43 | I_4_b | -226.0657526 | -377.5037816 |  | -0.014449 | -9.0668 |
| 44 | MICA_1_d | -379.8062111 | -531.2496068 |  | -0.019816 | -12.434 |
| 45 | MICA_4_a | -379.8062111 | -531.2499899 |  | -0.020199 | -12.675 |
| 46 | MIMO_6_a | -379.8006614 | -531.2476615 |  | -0.023420 | -14.696 |
| 47 | MIMO_7_d | -379.806211 | -531.2516805 |  | -0.021889 | -13.736 |
| 48 | MIMO_8_d | -379.8062111 | -531.2530141 |  | -0.023223 | -14.573 |

**Table S3.** The effective fraction of hydrogen bond energy predicted according to the critical points of bonding between different structures and H_2_O_2_. And the energy offset of all statistical hydrogen bond energies compared to all intermolecular forces.

| **H_2_O_2_** | **a *10^-2^** | ***10^-2^** | ***10^-2^** | ***10^-2^** | ***10^-2^** | **Total（hartree）** | **Total-a（hartree）** | **a/T** | **Total**  **(kcal/mol)** | **Δ*E***  **(kcal/mol)** | **Offset** |
| --- | --- | --- | --- | --- | --- | --- | --- | --- | --- | --- | --- |
| 1_AI_2_bc |  | 1.73 | 1.72 | 1.16 |  | 0.046113 | 0.046113 | **0.00%** | -9.5446 | -11.528 | 17.21% |
| 2_AI_3_b |  | 2.52 | 4.08 |  |  | 0.066026 | 0.066026 | **0.00%** | -13.987 | -17.032 | 17.88% |
| 3_AI_7_c |  | 2.19 | 1.68 |  |  | 0.038716 | 0.038716 | **0.00%** | -7.8944 | -10.554 | 25.20% |
| 4_BICA_1_d | 3.74 | 1.05 |  |  |  | 0.047879 | 0.010503 | **78.06%** | -9.9385 | -13.945 | 28.73% |
| 5_BICA_4_d |  | 3.54 | 1.55 |  |  | 0.050904 | 0.050904 | **0.00%** | -10.613 | -13.59 | 21.90% |
| 6_BICA_6_a | 3.73 | 1.13 |  |  |  | 0.048529 | 0.011273 | **76.77%** | -10.084 | -14.393 | 29.94% |
| 7_BICA_37_b |  | 0.856 | 1.78 | 1.16 |  | 0.037997 | 0.037997 | **0.00%** | -7.734 | -10.834 | 28.61% |
| 8_FH_1_d | 4.21 | 1.05 | 1.41 | 0.866 |  | 0.075358 | 0.033208 | **55.93%** | -16.068 | -19.665 | 18.29% |
| 9_FH_8_dd |  | 0.836 | 4.19 | 3.47 | 0.678 | 0.091731 | 0.091731 | **0.00%** | -19.721 | -16.900 | 16.69% |
| 10_FH_11_d |  | 2.23 | 0.885 | 0.988 | 1.45 | 0.055533 | 0.055533 | **0.00%** | -11.646 | -17.250 | 32.49% |
| 11_FH_14_cd | 3.85 | 0.566 | 1.99 | 3.09 |  | 0.094984 | 0.056525 | **40.49%** | -20.447 | -22.844 | 10.49% |
| 12_FH_16_ad | 3.90 | 0.677 | 1.24 |  |  | 0.058218 | 0.019174 | **67.07%** | -12.245 | -9.2919 | 31.78% |
| 13_FH_17_a | 3.14 | 3.14 | 2.37 | 0.735 |  | 0.093858 | 0.062428 | **33.49%** | -20.196 | -20.420 | 1.10% |
| 14_FI_1_d | 3.12 | 2.08 |  |  |  | 0.052007 | 0.020845 | **59.92%** | -10.860 | -15.143 | 28.29% |
| 15_FI_4_a | 3.65 | 0.91 |  |  |  | 0.045561 | 0.009101 | **80.02%** | -9.4214 | -13.799 | 31.72% |
| 16_FI_6_ad | 3.12 | 2.08 |  |  |  | 0.052023 | 0.020850 | **59.92%** | -10.863 | -15.143 | 28.26% |
| 17_FI_13_bd |  | 3.66 | 4.44 |  |  | 0.080943 | 0.080943 | **0.00%** | -17.314 | -18.904 | 8.41% |
| 18_FI_14_b | 3.88 | 3.64 |  |  |  | 0.075173 | 0.036370 | **51.62%** | -16.027 | -18.489 | 13.31% |
| 19_H_3_a | 3.58 | 3.57 | 9.00 |  |  | 0.080578 | 0.044728 | **44.49%** | -17.233 | -25.652 | 32.82% |
| 20_H_5_c | 4.51 | 1.08 | 1.14 |  |  | 0.067341 | 0.022253 | **66.95%** | -14.280 | -17.117 | 16.57% |
| 21_H_7_ac | 3.58 | 3.57 | 0.901 |  |  | 0.080543 | 0.044720 | **44.48%** | -17.225 | -21.528 | 19.99% |
| 22_H_9_b | 4.52 | 1.00 | 0.826 |  |  | 0.063468 | 0.018281 | **71.20%** | -13.416 | -15.461 | 13.23% |
| 23_H_11_a | 3.42 | 1.48 | 3.68 |  |  | 0.085782 | 0.051555 | **39.90%** | -18.394 | -21.097 | 12.81% |
| 24_H_13_c | 4.58 | 1.39 | 1.04 |  |  | 0.070133 | 0.024323 | **65.32%** | -14.903 | -17.467 | 14.68% |
| 25_HAIL_6_b |  | 1.39 | 3.51 |  |  | 0.048978 | 0.048978 | **0.00%** | -10.184 | -15.035 | 32.27% |
| 26_HAIL_8_b |  | 4.17 | 1.58 |  |  | 0.057464 | 0.057464 | **0.00%** | -12.077 | -16.288 | 25.86% |
| 27_HAIL_13_c | 5.42 | 2.80 |  |  |  | 0.082158 | 0.027975 | **65.95%** | -17.585 | -23.221 | 24.27% |
| 28_His_1_cd | 5.78 | 2.70 | 2.20 |  |  | 0.10676 | 0.048974 | **54.13%** | -23.073 | -16.975 | 35.92% |
| 29_His_3_ac | 3.95 | 1.97 | 1.26 | 2.03 |  | 0.092095 | 0.052549 | **42.94%** | -19.802 | -20.463 | 3.23% |
| 30_His_4_a | 5.17 | 3.34 |  |  |  | 0.085152 | 0.033434 | **60.74%** | -18.253 | -24.784 | 26.35% |
| 31_His_7_ac | 3.71 | 1.14 | 2.55 | 0.991 |  | 0.083839 | 0.046784 | **44.20%** | -17.960 | -27.009 | 33.50% |
| 32_His_8_acd | 4.25 | 2.06 | 1.24 | 2.11 |  | 0.096628 | 0.054099 | **44.01%** | -20.813 | -20.336 | -2.35% |
| 33_His_10_d |  | 3.32 | 0.983 | 1.48 | 0.583 | 0.063692 | 0.063692 | **0.00%** | -13.466 | -20.159 | 33.20% |
| 34_His_11_ad | 4.48 | 1.90 | 2.34 | 0.785 |  | 0.095052 | 0.050252 | **47.13%** | -20.462 | -21.266 | 3.78% |
| 35_His_18_a | 0.832 | 1.22 | 4.06 | 1.26 |  | 0.073707 | 0.065385 | **11.29%** | -15.700 | -18.990 | 17.32% |
| 36_IAA_4_d |  | 2.56 | 1.26 | 0.307 |  | 0.041245 | 0.041245 | **0.00%** | -8.4587 | -11.104 | 23.82% |
| 37_IAA_8_a | 2.82 | 1.73 | 1.11 |  |  | 0.056617 | 0.028416 | **49.81%** | -11.888 | -15.579 | 23.70% |
| 38_IAA_10_a | 2.09 | 1.85 | 1.75 |  |  | 0.056925 | 0.036035 | **36.70%** | -11.957 | -15.932 | 24.95% |
| 39_IAA_31_d | 2.79 | 1.81 | 1.55 |  |  | 0.061382 | 0.033528 | **45.38%** | -12.951 | -14.342 | 9.70% |
| 40_I_1_a | 4.02 | 8.38 |  |  |  | 0.048537 | 0.008381 | **82.73%** | -10.085 | -14.391 | 29.92% |
| 41_I_2_b |  | 1.26 | 2.43 |  |  | 0.036852 | 0.036852 | **0.00%** | -7.4786 | -8.9398 | 16.34% |
| 42_I_3_a | 4.06 | 0.996 |  |  |  | 0.050603 | 0.009965 | **80.31%** | -10.546 | -15.210 | 30.66% |
| 43_I_4_b |  | 1.03 | 2.24 |  |  | 0.032724 | 0.032724 | **0.00%** | -6.5578 | -9.0668 | 27.67% |
| 44_MICA_1_d |  | 2.65 | 1.05 |  |  | 0.037021 | 0.037021 | **0.00%** | -7.5163 | -12.434 | 39.55% |
| 45_MICA_4_a | 4.11 | 0.825 |  |  |  | 0.04938 | 0.008251 | **83.29%** | -10.273 | -12.675 | 18.95% |
| 46_MIMO_6_a | 4.04 | 0.853 |  |  |  | 0.048965 | 0.008530 | **82.58%** | -10.181 | -14.696 | 30.72% |
| 47_MIMO_7_d | 1.27 | 2.51 | 0.879 | 0.879 |  | 0.055412 | 0.042713 | **22.92%** | -11.619 | -13.736 | 15.41% |
| 48_MIMO_8_d | 2.06 | 0.825 | 1.93 |  |  | 0.048112 | 0.027510 | **42.82%** | -9.9906 | -14.573 | 31.44% |

**Table S4.** The His analogs statistical table of molecular conformation frequency and effective hydrogen bond energy ratio in each conformation obtained according to the results of molecular docking and quantum chemical calculation.

|  | **AI** |  | **BICA** |  | **F** |  | **FI** |  | **H** |  | **HAIL** |  |
| --- | --- | --- | --- | --- | --- | --- | --- | --- | --- | --- | --- | --- |
| **Count** | 44 |  | 47 |  | 48 |  | 50 |  | 50 |  | 46 |  |
| **All_a** |  | 0 |  | 1 |  | 4 |  | 3 |  | 6 |  | 0 |
| **a** |  | 0 |  | 1 |  | 3 |  | 2 |  | 4 |  | 0 |
| **ac** |  | 0 |  | 0 |  | 0 |  | 0 |  | 2 |  | 0 |
| **ad** |  | 0 |  | 0 |  | 1 |  | 1 |  | 0 |  | 0 |
| **acd** |  | 0 |  | 0 |  | 0 |  | 0 |  | 0 |  | 0 |
| **ab** |  | 0 |  | 0 |  | 0 |  | 0 |  | 0 |  | 0 |
| **abd** |  | 0 |  | 0 |  | 0 |  | 0 |  | 0 |  | 0 |
| **abc** |  | 0 |  | 0 |  | 0 |  | 0 |  | 0 |  | 0 |
| **a%** |  | 0 |  | 0.016 |  | 0.021 |  | 0.033 |  | 0.033 |  | 0 |
| **ac%** |  |  |  |  |  |  |  |  |  | 0.018 |  |  |
| **ad%** |  |  |  |  |  | 0.014 |  | 0.012 |  |  |  |  |
| **acd%** |  |  |  |  |  |  |  |  |  |  |  |  |
| **ab%** |  |  |  |  |  |  |  |  |  |  |  |  |
| **abd%** |  |  |  |  |  |  |  |  |  |  |  |  |
| **abc%** |  |  |  |  |  |  |  |  |  |  |  |  |
|  |  |  |  |  |  |  |  |  |  |  |  |  |
| **All_a%** |  | 0 |  | 0.016 |  | 0.035 |  | 0.046 |  | 0.051 |  | 0 |
|  | **His** |  | **IAA** |  | **I** |  | **MICA** |  | **MIMO** |  |  |  |
| **Count** | 49 |  | 49 |  | 47 |  | 49 |  | 48 |  |  |  |
| **All_a** |  | 7 |  | 6 |  | 9 |  | 4 |  | 2 |  |  |
| **a** |  | 2 |  | 6 |  | 9 |  | 4 |  | 2 |  |  |
| **ac** |  | 3 |  | 0 |  | 0 |  | 0 |  | 0 |  |  |
| **ad** |  | 1 |  | 0 |  | 0 |  | 0 |  | 0 |  |  |
| **acd** |  | 1 |  | 0 |  | 0 |  | 0 |  | 0 |  |  |
| **ab** |  | 0 |  | 0 |  | 0 |  | 0 |  | 0 |  |  |
| **abd** |  | 0 |  | 0 |  | 0 |  | 0 |  | 0 |  |  |
| **abc** |  | 0 |  | 0 |  | 0 |  | 0 |  | 0 |  |  |
| **a%** |  | 0.025 |  | 0.045 |  | 0.152 |  | 0.068 |  | 0.034 |  |  |
| **ac%** |  | 0.027 |  |  |  |  |  |  |  |  |  |  |
| **ad%** |  | 0.01 |  |  |  |  |  |  |  |  |  |  |
| **acd%** |  | 0.009 |  |  |  |  |  |  |  |  |  |  |
| **ab%** |  |  |  |  |  |  |  |  |  |  |  |  |
| **abd%** |  |  |  |  |  |  |  |  |  |  |  |  |
| **abc%** |  |  |  |  |  |  |  |  |  |  |  |  |
|  |  |  |  |  |  |  |  |  |  |  |  |  |
| **All_a%** |  | 0.07 |  | 0.045 |  | 0.152 |  | 0.068 |  | 0.034 |  |  |

**Table S5.** The prediction of different classes of hydrogen bond based on the bond critical point (BCP) and intermolecular force between different structures and H_2_O.

| **H_2_O** | **a**  ***10^-2^** | ***10^-2^** | ***10^-2^** | ***10^-2^** | **Total (hartree）** | **Total (kcal/mol)** |
| --- | --- | --- | --- | --- | --- | --- |
| 6_BICA_6_a | 2.71 | 1.13 |  |  | 0.038425 | -7.8296 |
| 12_FH_16_ad |  | 2.32 | 0.670 | 1.14 | 0.041389 | -8.4907 |
| 13_FH_17_a | 2.20 | 2.22 | 2.59 |  | 0.070063 | -14.887 |
| 15_FI_4_a | 2.49 |  |  |  | 0.024904 | -4.8133 |
| 16_FI_6_ad | 2.12 | 1.46 |  |  | 0.035746 | -7.2320 |
| 19_H_3_a | 3.59 | 1.92 |  |  | 0.055098 | -11.549 |
| 21_H_7_ac | 3.59 | 1.92 |  |  | 0.055111 | -11.552 |
| 23_H_11_a | 1.18 | 3.01 |  |  | 0.041848 | -8.5931 |
| 29_His_3_ac | 2.93 | 0.627 | 2.31 | 0.939 | 0.068112 | -14.452 |
| 30_His_4_a | 3.13 | 1.60 | 5.17 |  | 0.098921 | -21.325 |
| 31_His_7_ac | 3.83 | 0.710 | 2.79 |  | 0.073293 | -15.608 |
| 32_His_8_acd | 3.11 | 0.661 | 2.49 | 1.01 | 0.072783 | -15.494 |
| 34_His_11_ad | 3.81 | 4.63 |  |  | 0.084413 | -18.088 |
| 35_His_18_a |  | 0.830 | 0.803 | 3.06 | 0.046960 | -9.7335 |
| 37_IAA_8_a | 3.15 | 1.44 |  |  | 0.045840 | -9.4837 |
| 38_IAA_10_a | 2.89 | 1.25 |  |  | 0.041399 | -8.4930 |
| 40_I_1_a | 2.79 |  |  |  | 0.027903 | -5.4823 |
| 42_I_3_a | 2.79 |  |  |  | 0.027937 | -5.4899 |
| 45_MICA_4_a | 2.86 |  |  |  | 0.028592 | -5.6360 |
| 46_MIMO_6_a | 2.82 |  |  |  | 0.028221 | -5.5532 |

**Table S6.** DNA sequences used in this work.

| Sequences Name | Sequences (5'-3') |
| --- | --- |
| G4_a_ | GGGTGGGTGGGTGGG |
| G4_b_ | **CT**GGGTGGGTGGGTGGG |
| G4_c_ | GGGTGGGTGGGTGGG**TC** |
| G4_d_ | **CT**GGGTGGGTGGGTGGG**TC** |
| mut-G4_d_ | CTG**A**GTG**A**GTG**A**GTGGGTC |

**Table S7.** The catalytic cost performance of HRP.

|  | HRP | | | | |
| --- | --- | --- | --- | --- | --- |
| Manufacturer | Sigma-Aldrich | | Macklin | | |
| SKU | P6782-5MG | 77332-100MG | P815746-100mg | P815747-100mg | P815749-25mg |
| Pack Size (mg) | 5 | 100 | 100 | 100 | 25 |
| Price (€) | 112.14 | 243.10 | 36.09 | 29.79 | 23.62 |
| Molecular Weight | 44000 | | | | |
| Unit Price (€/g) | 2.24*10^4^ | 2.43*10^3^ | 3.61*10^2^ | 2.98*10^2^ | 9.45*10^2^ |
| Unit Price (€/mol) | 9.87*10^8^ | 1.07*10^8^ | 1.59*10^7^ | 1.31*10^7^ | 4.16*10^7^ |
| Unit Price of catalyst (€/mol _cat._) | 9.87*10^8^ | 1.07*10^8^ | 1.59*10^7^ | 1.31*10^7^ | 4.16*10^7^ |
| *k*_cat_ (s^-1^) | 50-800 | | | | |
| Price (€/mol _sub._)*^a^* | 1.97*10^7^~1.23*10^6^ | 2.14*10^6^~1.34*10^5^ | 3.18*10^5^~1.98*10^4^ | 2.62*10^5^~1.64*10^4^ | 8.31*10^5^~5.20*10^4^ |
| Average Price | 1.05*10^7^ | 1.14*10^6^ | 1.69*10^5^ | 1.39*10^5^ | 4.42*10^5^ |
| Brand Average Price | 5.81*10^6^ | | 2.50*10^5^ | | |

^a^The cost of catalyzing 1mole substrate at maximum catalytic efficiency

**Table S8.** The catalytic cost performance of CPDzyme.

|  | CPDzyme | | |
| --- | --- | --- | --- |
| Manufacturer | Xi'an Ruixi Biological Technology | Sangon Biotech | Genscript |
| Name | Hemin-NHS | bio-TTTTTTTTT/iPCLink/TGGGTGGGTGGGTGGG-NH_2_ | Lys-His-Arg-Arg-His |
| Pack Size | 100 mg | 1 mol | 4 mg |
| Price (€) | 590.55 | 7.31*10^9^ | 33.86 |
| Molecular Weight | 846.1 | / | 732.85 |
| Unit Price (€/g) | 5.91*10^3^ | / | 8.46*10^3^ |
| Unit Price (€/mol) | 5.00*10^6^ | 7.31*10^9^ | 6.20*10^6^ |
| Unit Price of Catalyst (€/mol _cat._) | 7.32*10^9^ | | |
| *k*_cat_ (s^-1^) | 784 | | |
| Price(€/mol _sub._) | 9.33*10^6^ | | |

**Table S9.** The catalytic cost performance of G4/hemin.

|  | G4/hemin | |
| --- | --- | --- |
| Manufacturer | Bide Pharmatech | Sangon Biotech |
| Name | hemin | TT GGG T GGG T GGG T GGG T |
| Pack Size | 25 g | 1 mol |
| Price (€) | 59.19 | 7.35*10^6^ |
| Molecular Weight | 651.94 | / |
| Unit Price (€/g) | 2.37 | / |
| Unit Price (€/mol) | 1.54*10^3^ | 7.35*10^6^ |
| Unit Price of Catalyst (€/mol _cat._) | 7.35*10^6^ | |
| *k*_cat_ (s^-1^) | 0.3 | |
| Price(€/mol _sub._) | 2.45*10^7^ | |

**Table S10.** The catalytic cost performance of AA-heminzyme and chemiluminescent nanomaterial.

|  | FhI |  |  | FG4_d_hI | | FG4_d_hIL | |  |
| --- | --- | --- | --- | --- | --- | --- | --- | --- |
| Manufacturer | Bide Pharmatech | | | Sangon Biotech | Bide Pharmatech | | | |
| Name | Fmoc-histidine | hemin | Imidazole | CTGGGTGGGTGGGTGGGTC | luminol | |  |  |
| Pack Size | 25 g | 25 g | 500 g | 1 mol | 25g | |  |  |
| Price (€) | 95.93 | 59.19 | 8.66 | 8.02*10^6^ | 21.39 | |  |  |
| Molecular Weight | 377.39 | 651.94 | 68.07 | / | 177.16 | |  |  |
| Unit Price (€/g) | 3.84 | 2.37 | 1.73*10^-2^ | / | 0.856 | |  |  |
| Unit Price (€/mol) | 1.45*10^3^ | 1.54*10^3^ | 1.18 | 8.02*10^6^ | 1.52*10^2^ | |  |  |
| Unit Price of Catalyst or chemiluminescent nanomaterial (€/mol _cat._) | 1.02*10^4^ |  |  | 8.03*10^6^ | | 8.03*10^6^ | |  |
| *k*_cat_ (s^-1^) | 35.1 |  |  | 115 | | / | |  |
| Price(€/mol _sub._) | 2.92*10^2^ |  |  | 6.89*10^4^ | | / | |  |

**Table S11.** The catalytic cost performance of different catalysts.

| Catalysts | HRP | CPDzyme | G4/hemin | FhI | FG4_d_hI |
| --- | --- | --- | --- | --- | --- |
| Price (€/mol_sub._) | 5.81*10^6^~2.50*10^5^ | 9.33*10^6^ | 2.45*10^7^ | 2.92*10^2^ | 6.89*10^4^ |

**Table S12.** Comparison of different methods for detection of GSH.

| Methods | Materials | Linear range (μM) | LOD (nM) | Ref. |
| --- | --- | --- | --- | --- |
| Colorimetry | ZnMn_2_O_4_-MtNCs | 10–700 | 520 | ^[1]^ |
|  | Fe-N/S-C SAzymes | 0.8-100 | 190 | ^[2]^ |
|  | Fe_3_O_4_/N-HCS | 0.02–250 | 9.3 | ^[3]^ |
| Fluorimetry | g-C_3_N_4_ | 0.2-45.0 | 140 | ^[4]^ |
|  | PDs-MnO_2_ | 0.5-200 | 100 | ^[5]^ |
|  | AuNCs@Lys-MnO_2_ | 0.5–1000 | 5 | ^[6]^ |
| Electro-  chemiluminescence | Ru(bpy)_2_(cpaphen)^2+^/  TPrA/TiO_2_ | 5-215 | 330 | ^[7]^ |
|  | g-C_3_N_4_–MnO_2_ | 0.2-100 | 50 | ^[8]^ |
|  | A-COF-MnO_2_ | 0.05 – 100 | 17 | ^[9]^ |
| Chemiluminescence | CoOOH NFs | 0.01-1 | 6.4 | ^[10]^ |
|  | FeS_2_NPs/MnO_2_NSs | 1–500 | 150 | ^[11]^ |
|  | FhG4_d_IL | 0.5-200 | 0.194 | This work |

**Table S13.** Determination of GSH in human serum samples (n = 3).

| Sample | GSH Content Assay Kit (mM) | POCT by smartphone (mM, n=3) | Deviation rate (%) |
| --- | --- | --- | --- |
| 1 | 1.71±0.024 | 1.57±0.055 | -8.19 |
| 2 | 1.59±0.006 | 1.61±0.098 | 1.26 |
| 3 | 1.05±0.09 | 1.12±0.12 | 6.67 |
| 4 | 0.85±0.051 | 0.88±0.078 | 3.53 |
| 5 | 1.67±0.043 | 1.75±0.330 | 4.79 |
| 6 | 1.35±0.085 | 1.39±0.065 | 2.96 |
| 7 | 1.62±0.019 | 1.71±0.152 | 5.56 |
| 8 | 0.71±0.011 | 0.70±0.115 | -1.41 |
| 9 | 1.40±0.025 | 1.45±0.065 | 3.57 |
| 10 | 1.25±0.12 | 1.31±0.081 | 4.8 |
| 11 | 1.12±0.056 | 1.16±0.048 | 3.57 |
| 12 | 0.73±0.041 | 0.70±0.085 | -3.70 |

The linear equation of GSH kit is y = 0.0052x + 0.1667 (R^2^ = 0.9998), and the detection range is 20-100uM. The linear equation of our POCT colorimetric method is y = -0.62 x + 73.33 (R^2^ = 0.99955), and the detection range is 5-100 µM.

**Table S14.** The cost comparison of GSH Content Assay Kit and POCT based on FG4_d_hIL-H_2_O_2_ for GSH detection.

|  | GSH Content Assay Kit | FG4hIL-H_2_O_2_ |
| --- | --- | --- |
| Manufacturer | Leagene | This work |
| Price (€) | 36.75 | / |
| Unit Price of chemiluminescent nanomaterial (€/mol) | / | 8.03*10^6^ |
| H_2_O_2_ (€/mol) | / | 5.17 |
| FG4hIL Concentration (μM) |  | 5 |
| H_2_O_2_ Concentration (mM) | / | 100 |
| Detection Volume (μL) | / | 200 |
| Price of one sample (€) | 0.37 | 8.13*10^-3^ |

**Supplementary References**

[1] H. Li, P. Song, T. Wu, H. Zhao, Q. Liu, X. Zhu, *Appl. Clay Sci.* **2022**, *229, 106656*.

[2] R. Li, X. He, R. Javed, J. Cai, H. Cao, X. Liu, Q. Chen, D. Ye, H. Zhao, *Sci. Total Environ.* **2022**, *834*, 155428.

[3] Y. Zheng, D. Xu, L. Sun, J. Ji, J. Sun, Z. Tong, L. Qin, Y. Zhang, J. Luo, D. Liao, *Colloids Surf. A Physicochem. Eng. Aspects* **2022**, *648*.

[4] C. Cheng, Y. Huang, J. Wang, B. Zheng, H. Yuan, D. Xiao, *Anal. Chem.* **2013**, *85*, 2601-2605.

[5] L. Han, S. G. Liu, X. F. Zhang, B. X. Tao, N. B. Li, H. Q. Luo, *Sens. Actuators B Chem.* **2018**, *258*, 25-31.

[6] W. Fu, H. Wang, Y. Chen, J. Ding, G. Shan, *Microchem. J.* **2020**, *159*.

[7] R. Zhang, X. Zhong, A. Y. Chen, J. L. Liu, S. K. Li, Y. Q. Chai, Y. Zhuo, R. Yuan, *Anal. Chem.* **2019**, *91*, 3681-3686.

[8] X. L. Fu, F. Hou, F. R. Liu, S. W. Ren, J. T. Cao, Y. M. Liu, *Biosens. Bioelectron.* **2019**, *129*, 72-78.

[9] L. Song, W. Gao, Q. Han, Y. Huang, L. Cui, C. Y. Zhang, *Chem. Commun.* **2022**, *58*, 10524-10527.

[10] X. J. Yang, R. S. Li, C. M. Li, Y. F. Li, C. Z. Huang, *Talanta* **2020**, *215*, 120928.

[11] X. Liu, Q. Fan, X. Zhang, M. Li, Y. Huan, P. Ma, D. Song, Q. Fei, *Talanta* **2022**, *240*, 123171.
